# Population genomic evidence for adaptive differentiation in Baltic Sea three-spined sticklebacks

**DOI:** 10.1186/s12915-015-0130-8

**Published:** 2015-03-24

**Authors:** Baocheng Guo, Jacquelin DeFaveri, Graciela Sotelo, Abhilash Nair, Juha Merilä

**Affiliations:** Department of Biosciences, Ecological Genetics Research Unit, University of Helsinki, PO Box 65, Helsinki, FI-00014 Finland; Current address: CIBIO - Centro de Investigação em Biodiversidade e Recursos Genéticos, InBIO – Laboratório Associado, Universidade do Porto, Vairão, 4485-661 Portugal; Current address: Department of Biosciences, Metapopulation Research Group, University of Helsinki, PO Box 65, Helsinki, FI-00014 Finland

**Keywords:** *Gasterosteus aculeatus*, RAD-sequencing, SNP, population differentiation, local adaptation, Baltic Sea

## Abstract

**Background:**

The degree of genetic differentiation among populations experiencing high levels of gene flow is expected to be low for neutral genomic sites, but substantial divergence can occur in sites subject to directional selection. Studies of highly mobile marine fish populations provide an opportunity to investigate this kind of heterogeneous genomic differentiation, but most studies to this effect have focused on a relatively low number of genetic markers and/or few populations. Hence, the patterns and extent of genomic divergence in high-gene-flow marine fish populations remain poorly understood.

**Results:**

We here investigated genome-wide patterns of genetic variability and differentiation in ten marine populations of three-spined stickleback (*Gasterosteus aculeatus*) distributed across a steep salinity and temperature gradient in the Baltic Sea, by utilizing >30,000 single nucleotide polymorphisms obtained with a pooled RAD-seq approach. We found that genetic diversity and differentiation varied widely across the genome, and identified numerous fairly narrow genomic regions exhibiting signatures of both divergent and balancing selection. Evidence was uncovered for substantial genetic differentiation associated with both salinity and temperature gradients, and many candidate genes associated with local adaptation in the Baltic Sea were identified.

**Conclusions:**

The patterns of genetic diversity and differentiation, as well as candidate genes associated with adaptation, in Baltic Sea sticklebacks were similar to those observed in earlier comparisons between marine and freshwater populations, suggesting that similar processes may be driving adaptation to brackish and freshwater environments. Taken together, our results provide strong evidence for heterogenic genomic divergence driven by local adaptation in the face of gene flow along an environmental gradient in the post-glacially formed Baltic Sea.

**Electronic supplementary material:**

The online version of this article (doi:10.1186/s12915-015-0130-8) contains supplementary material, which is available to authorized users.

## Background

While local adaptation is likely to be of commonplace occurrence, demonstrating its occurrence can be difficult and take substantial research efforts [1-3]. Traditionally, studies of local adaptation have been built upon quantitative genetic approaches that make use of common garden experiments and statistical genetics methods to infer genetic differentiation in phenotypic traits (e.g., [4-6]). Quantitative genetic methods have also been increasingly combined with population genetic tools to infer local adaptation (e.g., [7-10]). More recently, advances in genomic technologies have made it possible to identify candidate genomic regions underlying local adaptation (e.g., [11-14]). Among such approaches are genome scan or outlier detection methods (e.g., [15-17]), which allow inferences about adaptive differentiation to be made without the application of common garden experiments.

Outlier detection methods have become particularly popular in identifying population structuring and adaptive differentiation in marine fishes, which generally show very low levels of genetic differentiation in neutral marker genes [18-22] and in which common garden experiments are often logistically demanding, if not impossible, to conduct (but see [23-27]). However, with few notable exceptions (e.g., [28-32]), genome scan studies of marine fishes have typically been limited to tens – or in rare cases hundreds – of loci (e.g., [33-38]). As such, genome-wide patterns of diversity and divergence cannot thoroughly be explored, especially when the markers employed are anonymous. On the other hand, when high-throughput approaches have been used to screen thousands of loci across the genome, only a small number of populations have been under focus – generally those with obvious differentiation [29,39-41]. Hence, high-throughput population genomic studies aimed at detecting adaptive differentiation in marine fishes are rare, especially those employing a comprehensive sampling scheme. The latter point is particularly relevant in the context of seascape genetics, which aims to integrate environmental features with population genetic data to assess their impact on the genetic structure of marine populations [42,43]. In such approaches, sampling of multiple populations across environmental gradients becomes critical for inferences about genotype–environment associations.

There has been considerable interest in studying local adaptation and genetic differentiation in three-spined sticklebacks (*Gasterosteus aculeatus*; e.g., [25,44-54] and reviewed in [55]). However, most of these studies – particularly those using high-throughput methods [13,56-65] – have focused on marine–freshwater or lake–stream differentiation, with less focus on differentiation within the ancestral marine environment (but see [25,33,47,48,56,58,66]). Nevertheless, the studies thus far – irrespective of the approach used (common garden: [25]; *Q*_ST_-*F*_ST_: [48]; population genetics or genomics: [33,47,51,53,58]) – suggest that there is substantial population structuring and local adaptation also in marine three-spined sticklebacks (but see [56,60]). This is most apparent in the thoroughly studied Baltic Sea seascape, which is characterized by steep salinity and temperature gradients. Using microsatellite markers, DeFaveri et al*.* [47] uncovered evidence for heterogeneous genomic differentiation and adaptive population structuring in three-spined sticklebacks across the Baltic Sea, suggesting that environmental heterogeneity can generate disruptive selection despite the considerable gene flow in this highly connected marine environment. In fact, the unique ecosystem of the Baltic Sea has attracted the attention of many evolutionary and population genetics studies that have also sought to understand local adaptation and genetic structuring of Baltic Sea organisms (e.g., [35,36,67,68]; reviewed in [69]). However, as yet, studies based on genome-wide characterizations of variability with high-throughput approaches and comprehensive sampling of Baltic Sea populations are still missing (but see [29,31]). Hence, the spatial scale of genetic structuring at the genome-wide level cannot truly be defined in any Baltic Sea organism.

**Figure 1:**
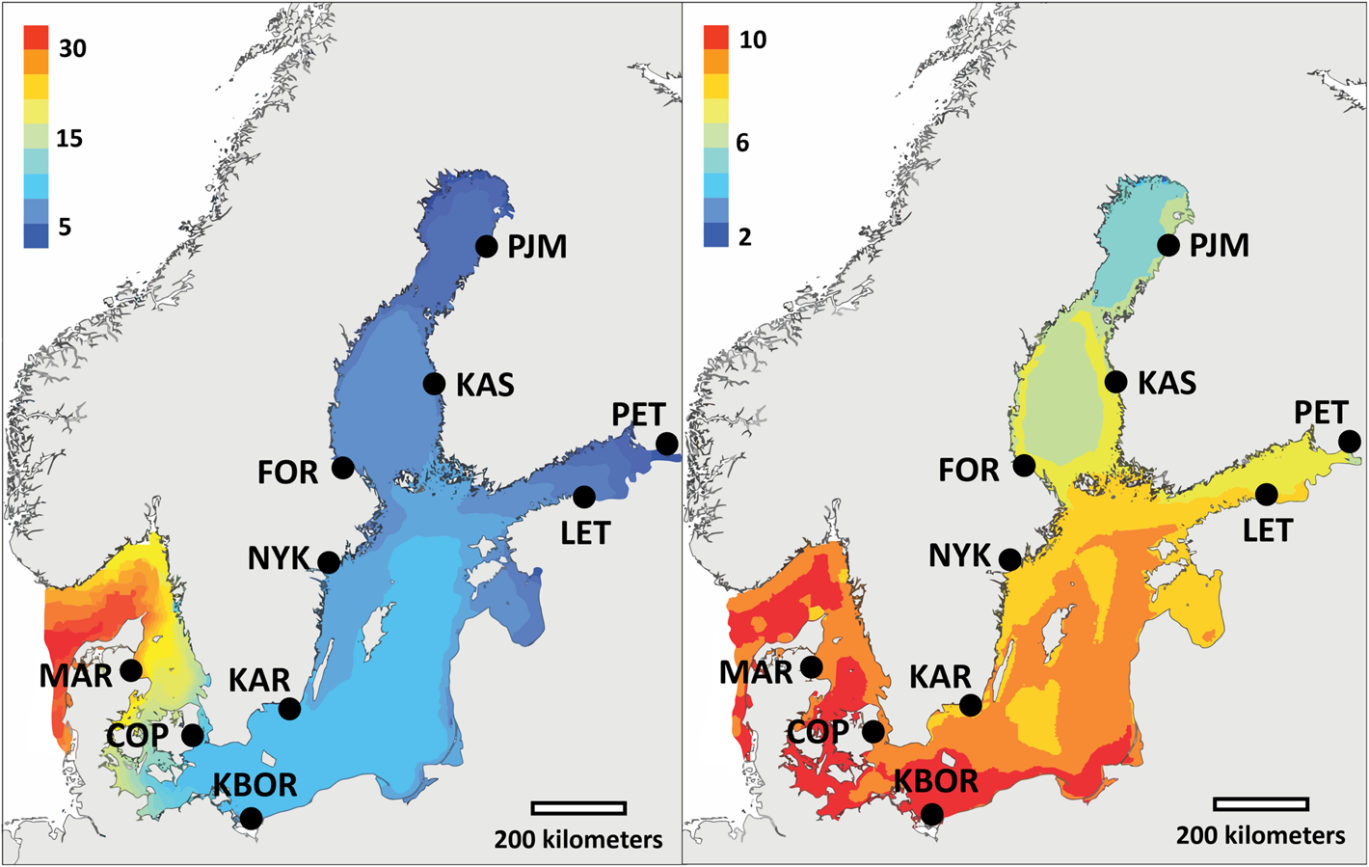
**Map showing the location of the study populations in the Baltic Sea area.** Left panel: Mean annual salinity (‰). Right panel: Mean annual temperature (°C). Adapted from [47].

The main aim of this study was to investigate genome-wide patterns of genetic variability and differentiation in marine three-spined sticklebacks across the Baltic Sea – a relatively young sea area with steep environmental gradients, subject to many earlier low-throughput studies in local adaptation (reviewed in [25,69]) and genetic differentiation (reviewed in [70,71]). In particular, we were interested in characterizing genomic variation across study sites that are connected by gene flow, and identifying genomic regions showing footprints of directional (and balancing) selection in association with key environmental parameters (viz*.* temperature and salinity). In addition, we were interested in knowing if the detected outliers correspond to those identified to be under selection in earlier stickleback studies, not only in this particular system [47] but also in other more divergent population pairs (e.g., [13,57,58,60,61,63-65]). To this end, we utilized a pooled restriction site associated DNA sequencing (RAD-seq) approach [72] to generate polymorphism data of >30,000 single nucleotide sites across the genome of 10 three-spined stickleback populations in the Baltic Sea (Figure 1 and Table 1), and subjected the data to various outlier analyses, including BAYENV [73], which tests for associations between outliers and environmental parameters.

**Table 1:** **Sampling information of populations used in this study**

**Location**	**Basin/region**	**Code**	**Coordinates**	**Average annual salinity (ppt)**	**Average annual temperature (°C)**	***N*** ^**a**^
Copenhagen	Kattegat	COP	55°42'05"N, 12°35'55"E	10.76	9.85	36
Forsmark	Bothnian Sea	FOR	60°24'09"N, 18°11'05"E	4.73	7.82	36
Karlskrona	Baltic Proper	KAR	56°10'13"N, 15°24'34"E	7.55	9.43	36
Kaskinen	Bothnian Sea	KAS	62°23'02"N, 25°54'52"E	3.15	7.51	36
Karsibor	Karsibor	KBOR	53°52'14"N, 14°17'03"E	7	9.69	36
Letipea	Gulf of Finland	LET	59°32'58"N, 26°35'49"E	4.63	7.80	36
Mariager Fjord	Kattegat	MAR	56°38'58"N, 09°57'05"E	26.82	9.88	36
Nyköping	Baltic Proper	NYK	58°39'04"N, 17°06'02"E	6.58	8.20	36
Petergofa	Gulf of Finland	PET	60°03'14"N, 29°58'16"E	0	7.42	36
Pyhäjoki	Bothnian Bay	PJM	64°28'42"N, 24°13'13"E	3.44	6.09	36

^a^ *N* is the number of individuals sequenced.

## Results

### Restriction site associated DNA sequencing dataset and SNPs

The three-spined stickleback genome used as a reference included 317,852 *Pst*I restriction sites (Additional file 1: Table S1). The number of *Pst*I restriction sites on a given chromosome was significantly correlated with chromosome length (*r*_*s*_ > 0.98, *P* < 1.71 × 10^−16^). The quality-filtered RAD-seq dataset used for alignment contained approximately 35.3 million reads, and the number of reads ranged between 2.4 and 4.4 million within each population (Additional file 1: Table S1). In total, 12.3 million reads were aligned to the reference genome, and the number of aligned reads ranged from 0.7 to 1.8 million within each population (Additional file 1: Table S1). The number of RAD-seq reads aligned on a given chromosome was significantly and positively correlated with chromosome length (*r*_*s*_ > 0.51, *P* < 0.01), both within and across populations. The number of SNPs within the aligned RAD-seq reads varied from 13,738 to 34,676 depending on the population, and in total 143,560 SNPs were identified across all populations (Additional file 1: Table S2). The number of SNPs identified on each chromosome was significantly and positively correlated with the number of reads aligned on the chromosome (*r*_*s*_ > 0.95, *P* < 2.2 × 10^−16^) and with chromosome length (*r*_*s*_ > 0.54, *P* < 0.01) within each population, as well across all the populations. An example using the population COP is shown in Additional file 2: Figure S1: the number of *Pst*I restriction sites, mapped reads and number of SNPs on each chromosome are each significantly positively correlated with chromosome length. Taken together, this suggests that the loci used in the downstream analyses are not a biased sample across chromosomes in respect to chromosome length.

### Genome-wide genetic variation

The expected heterozygosity for all SNPs (Table 2) across all populations was 0.29721 and ranged from 0.21416 to 0.25750 within each population. The genome-wide average nucleotide diversity (Tajima’s *π*) was 0.00358 (standard error (SE) = 0.00003) across all populations and ranged from 0.00284 to 0.00332 within each population (Table 2). Average *π* values of each chromosome within each population and across all populations are listed in Additional file 1: Table S3. The highest average *π* value within each population and across all populations was detected in chromosome XIX, which is the sex chromosome. Although the average nucleotide diversity in a given chromosome was significantly and positively correlated across populations (*r*_*s*_ > 0.56, *P* < 0.01), there was clear genomic heterogeneity in the levels of nucleotide diversity in different chromosomes. For example, the average *π* values for chromosomes III, VI, VII, VIII, IX, X, XI, XIV, XV, XX and XXI were larger than their genome-wide average *π* values in some populations, but smaller in other populations. The genome-wide average *θ*_*W*_ value was 0.00771 (SE = 0.00005) across all populations, and ranged from 0.00317 to 0.00403 within each population (Table 2).

**Table 2:** **Summary statistics of RAD data, including and estimates of basic population genetic parameters in each population**

**Population**	**Number of raw reads**	**Number of mapped reads**	**Number of SNPs** ^**a**^	**Average heterozygosity** ^**b**^	**Tajima’s** ***π***	**Watterson’s** ***θ***	**Proportion of SNPs** ^**c**^	**Number of private SNPs** ^**d**^
COP	3,751,563	1,220,231	22,850	0.23404	0.00303	0.00367	0.61313	64
FOR	3,548,488	1,167,788	21,196	0.22072	0.00301	0.00378	0.61854	51
KAR	3,490,058	1,765,802	34,676	0.25750	0.00275	0.00317	0.59648	71
KAS	3,875,084	1,665,176	29,022	0.22843	0.00286	0.00347	0.65246	107
KBOR	3,513,975	1,449,015	25,849	0.24355	0.00284	0.00336	0.60546	84
LET	3,452,354	789,771	15,746	0.23002	0.00332	0.00408	0.55612	21
MAR	3,419,749	1,532,462	29,957	0.23845	0.00300	0.00361	0.61900	118
NYK	2,418,564	696,118	13,738	0.23560	0.00332	0.00403	0.52402	8
PET	4,379,234	1,221,772	21,503	0.21416	0.00307	0.00391	0.62023	59
PJM	3,454,537	803,583	14,183	0.22936	0.00311	0.00380	0.55930	35

^a^SNPs identified using PoPoolation, used for average estimation of heterozygosity, Tajima’s *π*, and Watterson’s *θ*.

^b^Average heterozygosity is the sum of [2 × *p* × (1 – *p*)] for all SNPs with the total number of all SNPs identified in each population, where *p* is the frequency of the most common allele.

^c^Proportion of SNPs that have read support for both alleles in each population in the total 30,871 SNPs identified using PoPoolation2.

^d^Number of SNPs that are unique to each population in the total 30,871 SNPs identified using PoPoolation2.

**Figure 2:**
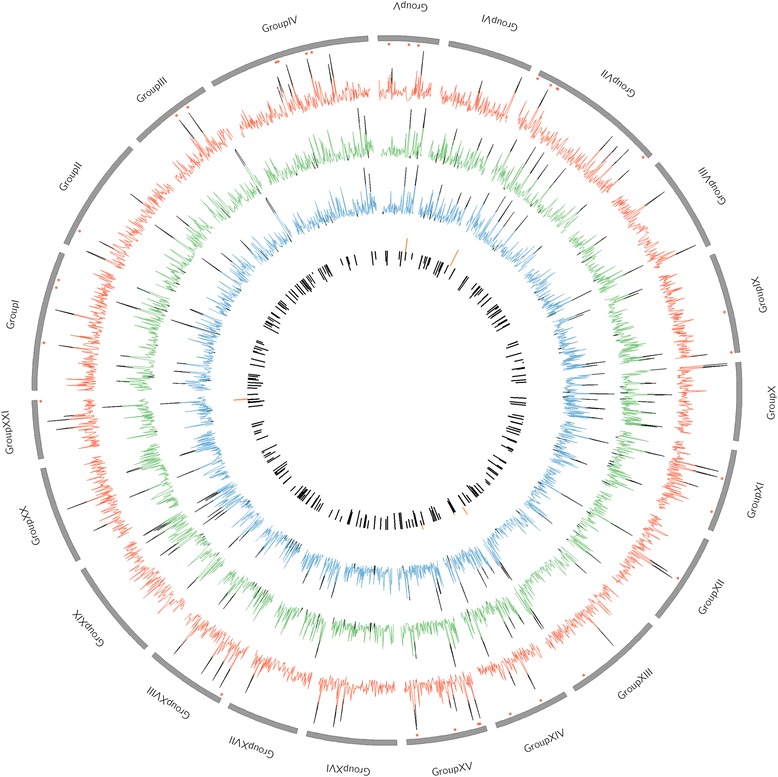
**Genome-wide distribution of genetic variation and differentiation across all the ten Baltic Sea three-spined stickleback populations.** Chromosomes are labeled in black Roman numerals and represented as grey blocks in a circle. The fixation index *F*_ST_ (red line), nucleotide diversity *π* (green line), population mutation rate *θ*_*W*_ (blue line) and Tajima’s *D* (black and orange histogram) are plotted as functions of genomic position with a non-overlapping 100-kb sliding window. The top 2% *F*_ST_, high *π*, and high *θ*_*W*_ and bottom 2% *π* and low *θ*_*W*_ are highlighted in black. Black and orange histograms represent Tajima’s *D* with negative and positive values, respectively. The red dots represented genomic regions with outlier loci.

The genome-wide distribution patterns for *π* and *θ*_*W*_ were very similar to each other across all populations (Figure 2), as well as within each population (Additional file 3: Figure S2), in respect to mean values of *π* and *θ*_*W*_ for each chromosome (*r*_*s*_ > 0.88, *P* < 2.65 × 10^−8^). However, the range of *π* and *θ*_*W*_ values varied widely across the genome across all populations and also within each population. For instance, *π* ranged from 0.00001 to 0.03038 and *θ*_*W*_ from 0.00017 to 0.03015 with the 100-kb sliding window (Figure 2). However, the genomic regions with high or low diversity were not consistent among populations (Additional file 3: Figure S2), suggesting genetic differentiation among populations.

To test for deviations from neutrality across all populations, Tajima’s *D* was estimated for 261 regions across the genome across all populations, of which 255 were negative (suggestive of positive or purifying selection) and six were positive (suggesting balancing selection; Figure 2). This observation of most genomic regions having negative Tajima’s *D* was found also within each population (Additional file 3: Figure S2). These patterns suggest an excess of low frequency variants.

### Genome-wide population differentiation

With a minor allele count of 4 across all the 10 populations and coverage between 10 and 500 within each population, 30,871 SNPs were identified by PoPoolation2. Pairwise *F*_ST_ values for the 30,871 SNPs estimated with a non-overlapping 100-kb sliding window across the genome yielded an overall average pairwise *F*_ST_ estimate of 0.02825 (SE = 0.00035) across all populations (range = 0.00178 to 0.27074; median = 0.02451). Comparison of the genome-wide profile of genetic differentiation (Figure 2) and diversity (Figure 2, Additional file 3: Figure S2) revealed certain general patterns. First, multiple genomic regions with high genetic diversity displayed low genetic differentiation (Figure 2), suggesting a role for balancing selection in maintaining high genetic diversity within and among populations. Inspection of the Tajima’s *D* estimates gave additional evidence for presence of balancing selection in genomic regions with elevated diversity: *D*-values were positive in genomic regions with high genetic diversity but low genetic differentiation (Figure 2). Second, numerous genomic regions on all chromosomes (except XIX) showing low genetic diversity exhibited a high degree of genetic differentiation (Figure 2), suggesting a varying degree of directional selection among populations.

### Candidate genes associated with adaptation

**Figure 3:**
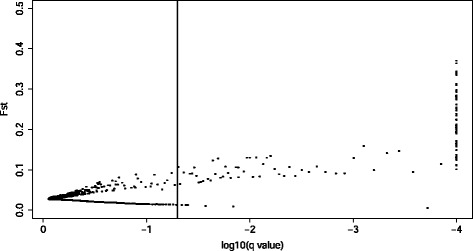
**Global outlier detection among the subset of 9,404 SNPs in 10 three-spined stickleback populations from the Baltic Sea.** The vertical line represents a false discovery threshold of 0.05.

In total, a subset of 9,404 SNPs located in 1,879 genes were identified across all populations (see Methods for criteria), and were used for detecting selection footprints. Using an empirical outlier detection approach, 530 (5.64%) SNPs were found at least once in the top 0.5% tails and 112 SNPs fell within the top 0.5% tails of at least 5 pairwise *F*_ST_ comparisons and as such, were identified as potential SNPs under selection. Using the BayeScan approach, 136 SNPs were identified as outliers (130 directionally selected and 6 under balancing selection; Figure 3) at the false discovery rate (FDR) threshold of 0.05. In total, 94 SNPs were identified as outliers by both approaches, all of which were under directional selection and located in genomic regions of high genetic differentiation (Figure 2). Of these 94 outlier loci, 74 (79%) were located within 26 genes (Additional file 1: Table S4).

**Figure 4:**
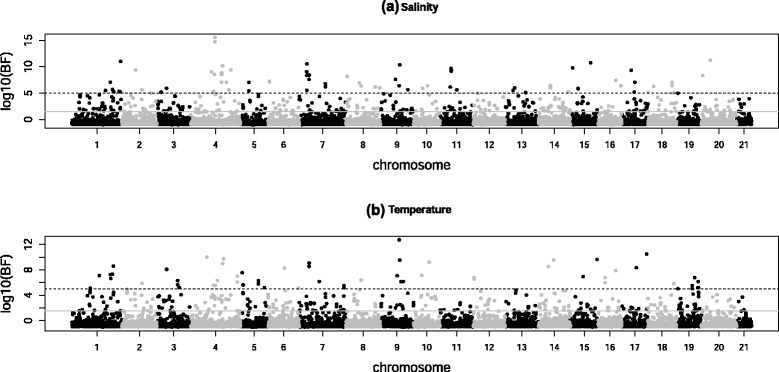
**Manhattan plot of genetic differentiation associated with environmental parameters.** It shows the SNP allele frequency variation associated with variation in **(a)** annual salinity and **(b)** annual temperature across different chromosomes. Grey solid lines mark lower thresholds of log_10_ (BF) = 1.5 and black dashed lines mark higher thresholds of log_10_ (BF) = 5.

The BAYENV analysis based on the subset of 9,404 SNPs revealed that both environmental parameters (salinity and temperature) were associated with numerous SNPs across the genome (Figure 4). With the criterion of log_10_ Bayes factor (BF) greater than 1.5 [74] as evidence for an association between environmental parameter and allelic distribution, 432 SNPs were correlated with variation in salinity (259 of which were located in 204 genes; Additional file 1: Table S4), and 413 SNPs were correlated with temperature variation (243 of which were located in 179 genes; Figure 4 and Additional file 1: Table S4). Moreover, 161 SNPs were significantly associated with variation in both salinity and temperature. When considering the correlation with salinity variation, 89 SNPs had log_10_ (BF) > 5 and 70 occurred in genomic regions with a high degree of genetic differentiation but low genetic diversity. The 89 loci were located in 39 genes; the SNP within the gene *CPEB4* (ENSGACG00000018422) on chromosome IV had the highest log_10_ (BF) (Figure 4a). When considering correlation with annual temperature variation, 73 loci had log_10_ (BF) > 5 and 49 occurred in genomic regions with a high degree of genetic differentiation but low genetic diversity. The 73 loci were located in 35 genes; the SNP within the gene *SMAP1* (ENSGACG00000018297) on chromosome IX had the highest log_10_ (BF) (Figure 4b). Of the 432 SNPs significantly associated with annual salinity variation, 45 were identified as outliers by BayeScan. Of the 413 SNPs significantly associated with annual temperature variation, 56 were identified as outliers by BayeScan. Of the 161 SNPs significantly associated with both salinity and temperature variation, 26 were identified as outliers by BayeScan; 14 of these were located in 11 genes.

**Figure 5:**
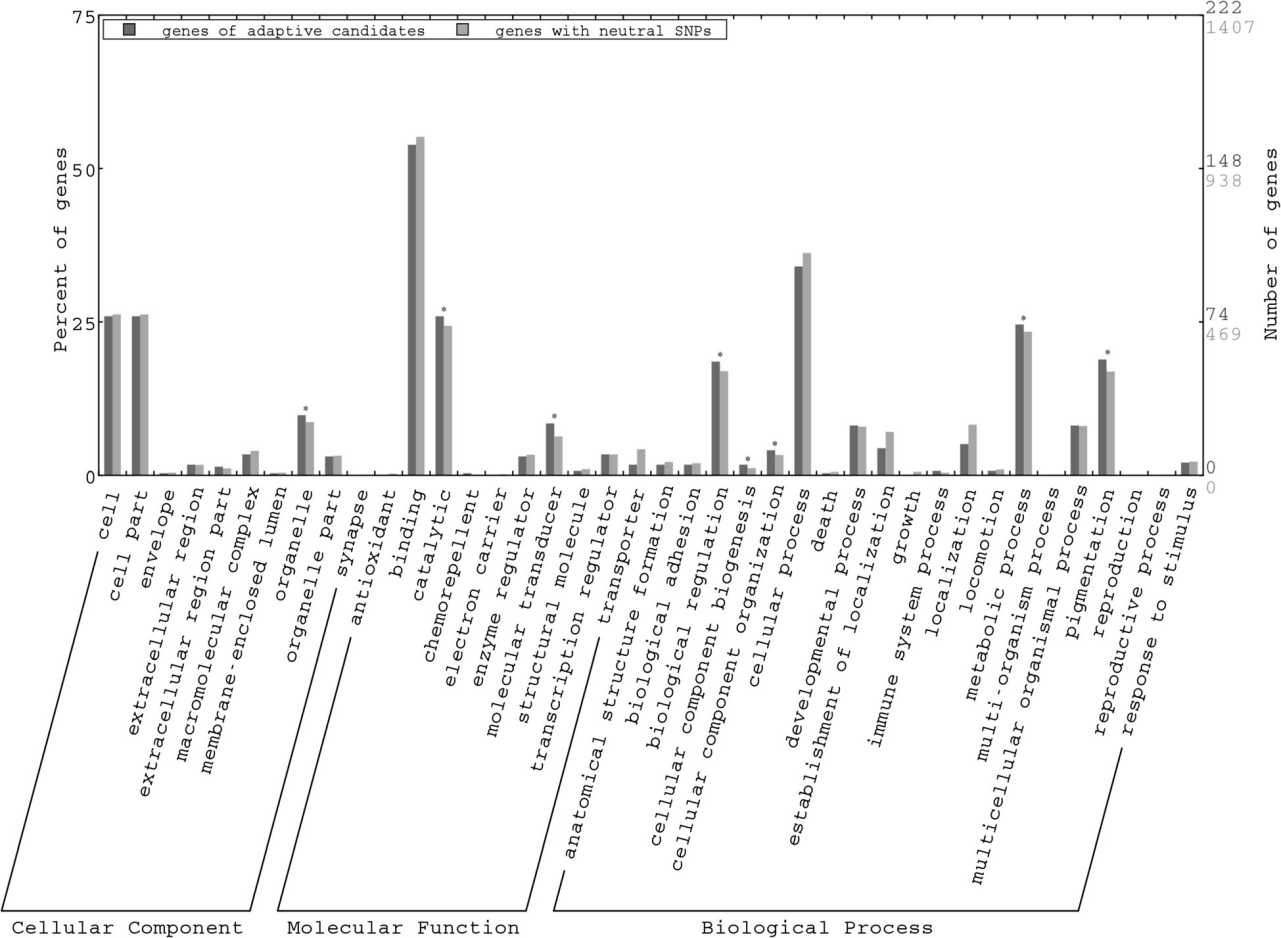
**Gene ontology assignment plot.** The plot shows GO of candidate genes for adaptive differentiation (containing outlier SNPs) and genes with neutral SNPs obtained with WEGO [120]. Asterisks indicate significantly enriched gene ontology terms (*P* < 0.05).

A total of 297 genes included SNPs that were identified either as outliers or as having environmental correlations in the BAYENV analysis (Additional file 1: Table S4). These candidate genes showed a broad range of gene ontology (GO) annotations, and significant enrichment in several functional categories (metabolic process, catalytic activity, organelle, pigmentation and signal transduction) when compared to the genes harboring neutral SNPs (*P* < 0.05, Figure 5).

### Population structure

**Figure 6:**
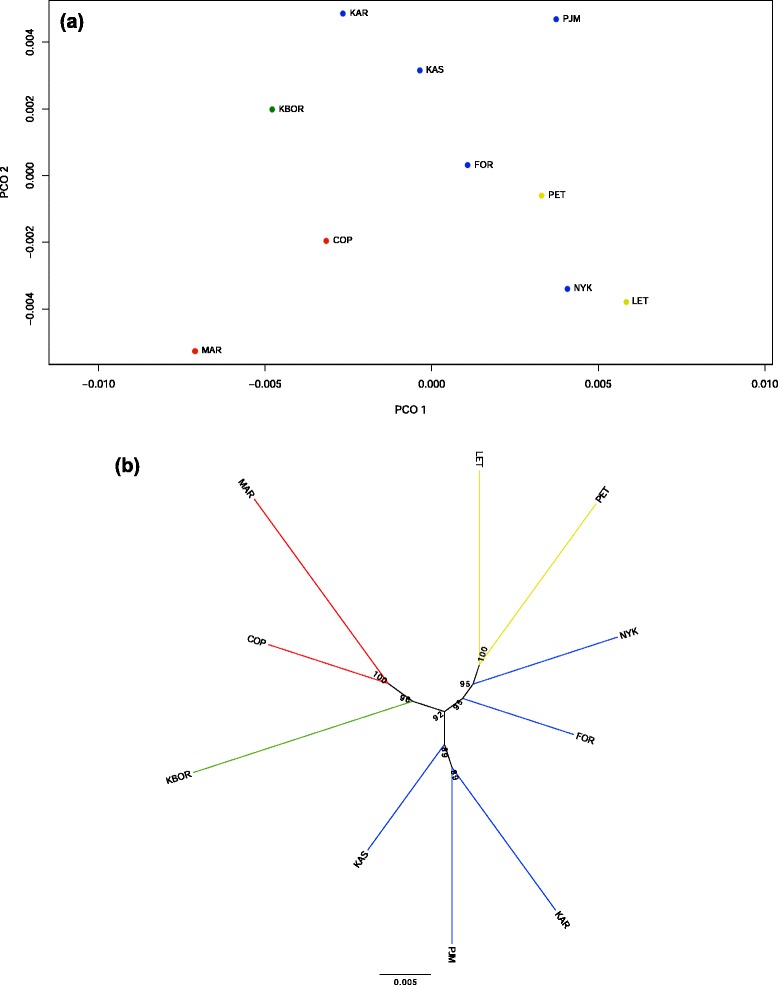
**Genetic relationships among the 10 three-spined stickleback populations from the Baltic Sea. (a)** Principal coordinates analysis (PCoA) plot of the overall average pairwise *F*_ST_ values of the subset of 9,404 SNPs among the 10 three-spined stickleback populations. **(b)** Neighbor-joining tree of the same populations based on *F*_ST_ values of each of the subset of 9,404 SNPs. Numbers on tree nodes represent bootstrap values of 1,000 replicates. Populations from different geographic regions are marked in color: red, North Sea; green, southern Baltic Sea; yellow, Gulf of Finland; blue, Gulf of Bothnia and Baltic proper.

We first took the overall average pairwise *F*_ST_ values among populations calculated over the subset of the 9,404 SNPs to look for population structuring across the Baltic Sea. The average pairwise *F*_ST_ values ranged from 0.00864 to 0.01548 (Table 3). The principal coordinates analysis (PCoA) plot revealed a geographically ordered pattern: the populations from the Danish Straits (MAR and COP) formed one distinct group and clustered close to the southern Baltic site KBOR along PCO 1; populations from the Gulf of Bothnia (KAS and PJM) and one from the south-west Baltic Sea (KAR) formed one cluster along PCO 2, and the populations from the Gulf of Finland (LET and PET) and Baltic Proper (FOR and NYK) clustered together (Figure 6a). The population structure portrayed by the neighbor-joining tree was very similar to that seen in the PCO plot of overall average pairwise *F*_ST_, but showed that populations from the Gulf of Finland (LET and PET) formed a distinct group (Figure 6b). This pattern of population structuring is consistent with that recovered by an earlier microsatellite study by DeFaveri et al. [47]. Accordingly, there was a significant correlation between pairwise genetic distances as measured by *F*_ST_ estimated from 40 microsatellites and the 9,404 SNPs (*r* = 0.46, *P* = 0.022, Mantel’s test). However, genetic diversity across the populations as estimated from microsatellite and SNP heterozygosity (or *π*) was uncorrelated (*r*_*s*_ < 0.25, *P* > 0.25). A signal of isolation by distance was detected (*r* = 0.41, *P* = 0.004, Mantel’s test).

**Table 3:** **Average pairwise** ***F***_**ST**_ **among ten Baltic Sea three-spined stickleback populations based on the subset of 9,404 SNPs**

	**COP**	**FOR**	**KAR**	**KAS**	**KBOR**	**LET**	**MAR**	**NYK**	**PET**	**PJM**
COP	–	–	–	–	–	–	–	–	–	–
FOR	0.00902	–	–	–	–	–	–	–	–	–
KAR	0.01139	0.01097	–	–	–	–	–	–	–	–
KAS	0.01064	0.00864	0.01011	–	–	–	–	–	–	–
KBOR	0.01060	0.01093	0.01142	0.01089	–	–	–	–	–	–
LET	0.01280	0.01112	0.01389	0.01251	0.01470	–	–	–	–	–
MAR	0.01018	0.01240	0.01291	0.01278	0.01233	0.01491	–	–	–	–
NYK	0.01182	0.01027	0.01347	0.01230	0.01365	0.01102	0.01391	–	–	–
PET	0.01176	0.00938	0.01340	0.01059	0.01279	0.01169	0.01439	0.01194	–	–
PJM	0.01210	0.00992	0.01104	0.01032	0.01307	0.01234	0.01548	0.01142	0.01193	–

## Discussion

Amongst the most salient findings of this study was the observation that although the average levels of genetic differentiation among Baltic Sea three-spined stickleback populations were low by all standards, numerous genomic regions displayed a high degree of population differentiation. Specifically, by utilizing stringent quality and filtering criteria, we identified numerous SNPs likely to have diverged due to directional selection, often apparently in response to either salinity- and/or temperature-mediated selection. Moreover, we also detected several genomic regions likely to be under balancing selection (i.e. regions showing high diversity and low divergence), including genomic regions harboring genes important for immune function. Interestingly, the patterns of genome-wide genetic variation and differentiation, as well as several candidate genes for local adaptation detected in this study, were similar to those observed in the marine–freshwater divergence of three-spined sticklebacks on various geographic scales [13,57,60,61,65], despite that the samples used in our study were derived from physically connected marine populations across the Baltic Sea. In the following, we will discuss these findings and relate our results to those of earlier studies of three-spined sticklebacks and other marine organisms. We will also discuss the implications of our findings for our understanding of local adaptation and genetic biodiversity in the Baltic Sea environment.

### Genome-wide heterogeneous differentiation in Baltic Sea three-spined stickleback

The patterns of genetic diversity and differentiation varied widely across the ten populations in the current study, indicating heterogeneous genomic divergence and divergent selection in Baltic Sea three-spined sticklebacks. These results are fully concurrent with those of an earlier study of Baltic Sea three-spined sticklebacks utilizing a much more limited number of microsatellite markers [47]. Although this kind of heterogeneous divergence has also been demonstrated in other studies of marine fish populations [75], to date very few such studies have employed high-throughput population genomic approaches – nor have they had access to reference genomes – to characterize the genomic architecture of adaptation (but see [28-31,58]). In this regard, we were able to provide a more refined picture of the genome-wide distribution of regions of differentiation in this open system of populations likely to experience multidirectional gene flow. Specifically, outlier loci/regions of divergence were uncovered on every chromosome, indicating that directional selection is acting across the entire genome, rather than being restricted to a few chromosomes, as was the case for the Atlantic cod [28]. Furthermore, many divergent genomic regions showed associations with salinity and temperature, supporting the interpretation that much of this differentiation could be driven by spatially varying selection pressures. As the use of pooled samples does not allow for investigations of linkage disequilibrium, we cannot ascertain the degree or distance of linkage between these regions of divergence. However, these isolated genomic regions usually span less than 100 kb in length, and the 94 outlier loci we identified were actually located in 38 different 100-kb sliding windows. Hence it is likely that individual loci within these regions are tightly linked, creating islands of divergence (*cf.* [76]) in the midst of the otherwise low neutral baseline divergence. This pattern of divergence hitchhiking is consistent with theoretical [77] and empirical [76,78-82] studies of populations experiencing ongoing gene flow (see also [83]). Moreover, the evidence for heterogeneous genomic divergence at the genome-wide level from this study aligns with the results of our earlier study that found a clear signal of isolation by adaptation, suggesting adaptive divergence has been reducing gene flow at a genome-wide scale [47]. As such, our results support the view (e.g., [28,47,84]) that selection is able to overcome the homogenizing effect of gene flow even in high-gene-flow marine environments.

Several regions of reduced divergence were also uncovered in this study. However, only a few of these regions gave evidence for balancing selection – far less than those of directional selection. In this respect, our results are in stark contrast with those of an early genome scan study of this species, which found evidence for the predominant role of balancing, rather than directional selection, on expressed sequence tag and quantitative trait locus associated microsatellite loci [85,86]. These contrasting results may be explained because highly mutable – and hence also polymorphic – microsatellites may generate spurious signals of balancing selection [85,87], whereas SNPs with lower mutation rates are less likely to generate such biases (*cf.* [88]).

### Candidate genes for adaptation

Our results show that three-spined sticklebacks in the Baltic Sea exhibit similar patterns of genetic differentiation and diversity as seen in earlier comparisons of marine–freshwater populations across their global distribution. For example, the distribution of diversity was similar to earlier studies that also reported elevated and lowered levels of genetic diversity at the ends and centers, respectively, of chromosomes I, III, XIII, XVIII and XX [58,60]. Not surprisingly, these regions of increased diversity also exhibited a lowered degree of divergence and a higher incidence of balancing selection compared to other parts of the genome. For instance, as in other stickleback studies [58,60], evidence for balancing selection was detected for the 3' end of chromosome III. This genomic region harbors several candidate genes involved in defense against pathogens (ENSGACG00000017648, ENSGACG00000017778 and ENSGACG00000017779), inflammation pathways (ENSGACG00000017812, ENSGACG00000017834 and ENSGACG00000017927), as well as TRIM genes (ENSGACG00000014250, ENSGACG00000014251, ENSGACG00000014283 and ENSGACG00000014403), which are known targets of balancing selection in primates [89], suggesting the importance of this genomic region for immune responses [60]. There is growing evidence from various vertebrate studies to suggest that genetic diversity in genes related to immunity is elevated and under balancing selection presumably due to their importance for many biological functions, including immunity, mate selection and kin recognition (reviewed in [90,91]). Since pathogens are strong selective agents [92] and the diversity and prevalence of stickleback parasites in the Baltic Sea is known to be high [93,94], the observed footprints of balancing selection on immunity-related genes are understandable.

Several genomic regions of reduced diversity and increased divergence detected in this study are also consistent with those reported in earlier studies of differentiation among marine–freshwater three-spined sticklebacks [49,60,65], as well as sticklebacks in the Baltic Sea [47]. For example, genomic regions on chromosomes I, IV, XI and XXI have repeatedly been identified as divergent between marine and freshwater three-spined sticklebacks in North America [60] and Eurasia [57,65]. Chromosome IV is of particular interest, as the gene related to lateral plate number variation – *Ectodysplasin A* (*eda*) [46] – is located within a genomic region of increased divergence on this chromosome (Figure 2). Our finding of elevated divergence in the genomic region containing the *eda* locus matches well with results of an earlier study that reported significant differentiation among Baltic Sea three-spined sticklebacks in both the number of lateral plates and the quantitative trait locus tightly linked to *eda* [48]. Thus, the results of our study provide matching evidence for ongoing selection on *eda* in Baltic Sea three-spined sticklebacks, and act as a proof-of-principle demonstration that the uncovered signatures of selection are likely to be real, rather than methodological artifacts or noise. The same is true for the gene *ahr1b (2 of 2)* (ENSGACG00000015615; Additional file 1: Table S4), which was identified as a candidate gene both in the current study as well as in our earlier genome scan of Baltic Sea sticklebacks [47]. Further evidence to substantiate this interpretation is provided by comparison to earlier targeted genome scan studies based on microsatellite markers in sticklebacks. These studies provided evidence for directional selection on 17 genomic regions harboring genes physiologically relevant for freshwater adaptation in a global survey of marine–freshwater populations [49], and for nine genomic regions in Baltic Sea populations [47]. The average pairwise *F*_ST_ for the genomic regions harboring these markers in this study was 0.033, which was slightly higher than the average *F*_ST_ across the whole genome (0.028), demonstrating that the regions harboring genes indicated to be under directional selection in earlier studies also show increased genomic divergence in the current study.

To set our results more firmly in the context of earlier targeted genome scan studies [33,47,49] and genome-wide sequencing studies [13,57,60,61,65], we compiled a list of candidate genes (i.e. genes containing outlier SNPs and/or SNPs associated with environmental variation) detected in our study (Additional file 1: Table S4) and compared these to those found in earlier studies. Of the 297 candidate genes identified here, 15 (5%) were also identified to be involved in marine–freshwater divergence of three-spined sticklebacks in earlier studies (Table 4), for example, genes *cpeb4* (cytoplasmic polyadenylation element binding protein 4, ENSGACG00000018422) and *pparaa* (peroxisome proliferator-activated receptor alpha a, ENSGACG00000018958) on chromosome IV [13,60,61]. An additional 22 were identified in a study investigating differential expression of salinity-related genes among freshwater and seawater sticklebacks acclimated to different salinity treatments [95]. Interestingly, allelic variation in most of these candidate genes (e.g., *cpeb4*) was strongly associated with salinity variation, suggesting that environmental salinity has been the selective agent driving genetic differentiation in these loci among Baltic Sea three-spined populations. In addition to the genes listed in Table 4, different paralogs from the same gene families were identified to be under selection both in the Baltic Sea and other three-spined stickleback populations. For example, the gene *slc6a17 (2 of 2)* (solute carrier family 6, member 17; ENSGACG00000007913) was significantly associated with annual salinity variation in Baltic Sea three-spined sticklebacks, whereas its highly similar paralog *slc6a3 (2 of 2)* (solute carrier family 6, member 3; ENSGACG00000018983) has been identified as a candidate for marine–freshwater divergence [60]. This suggests that some of the candidate genes that contribute to repeated adaptation of three-spined sticklebacks to freshwater habitats may also be involved with local adaptation in the environmentally heterogeneous Baltic Sea environment. However, it is worth noting that many candidate genes – and also the general patterns of diversity and divergence – identified in marine–freshwater population pairs are also found among pairs of lake–stream sticklebacks [63,96]. Hence, this may instead indicate that similar constraints imposed by the architecture of the stickleback genome generate similar patterns between our and earlier marine–freshwater studies, rather than similar processes (i.e. salinity-mediated selection). Moreover, it is important to note that in spite of the various lines of evidence that selection is acting on specific genes, empirical demonstration of their functional role is necessary ultimately to validate the inference of selection on candidate variants.

**Table 4:** **Representative list of genes that have been identified as candidates for adaptation**

**Ensembl gene ID**	**Gene name**	**Gene description**	**BayeScan**	**BAYENV**		**References**
			**Outlier** ^**a**^	**Salinity** ^**b**^	**Temperature** ^**b**^	
ENSGACG00000018422	*cpeb4*	Cytoplasmic polyadenylation element binding protein 4	Yes	**	*	[13,61,65]
ENSGACG00000018320	*flt4*	fms-related tyrosine kinase 4	–	**	**	[60,61]
ENSGACG00000007263	*pde4ba*	Phosphodiesterase 4B, cAMP-specific a	–	**	*	[13,60]
ENSGACG00000015515	*pde4ca*	Phosphodiesterase 4C, cAMP-specific a	–	*	–	[13]
ENSGACG00000001583	*pex5*	Peroxisomal biogenesis factor 5-Like	–	*	–	[13]
**ENSGACG00000018958**	***pparaa***	**Peroxisome proliferator-activated receptor alpha a**	**Yes**	**	*	[13,60,65,96]
ENSGACG00000008634	*stat5.1*	Signal transducer and activator of transcription 5.1	–	**	–	[13,60,61,65]
ENSGACG00000014429	*zgc:85722*	Family with sequence similarity 184, member A	–	*	**	[60,61]
**ENSGACG00000009393**	***FAM19A1***	**Family with sequence similarity 19 (chemokine (C-C motif)-like), member A1**	**–**	**–**	*	[57,95]
ENSGACG00000002723	*pi15a*	Peptidase inhibitor 15a	–	*	–	[60,65]
ENSGACG00000007629	*acer1*	Alkaline ceramidase 1	–	**	**	[60]
ENSGACG00000008897	*STAC2 (2 of 2)*	SH3 and cysteine rich domain 2	–	**	–	[60]
**ENSGACG00000019342**			**–**	**–**	*	[60,95]
ENSGACG00000020327	*aifm1*	Apoptosis-inducing factor, mitochondrion-associated 1	–	*	*	[60]
ENSGACG00000017985	*ctnna2*	Catenin (cadherin-associated protein), alpha 2	–	–	*	[65]
**ENSGACG00000000827**	***pygmb***	**Phosphorylase, glycogen (muscle) b**	**–**	*	*	[95]
**ENSGACG00000001963**	***enpep***	**Glutamyl aminopeptidase**	**–**	**–**	*	[95]
**ENSGACG00000002497**	***fam65a***	**Family with sequence similarity 65, member A**	**–**	**–**	*	[95]
**ENSGACG00000003512**	***crb2b***	**Crumbs homolog 2b**	**–**	*	**–**	[95]
**ENSGACG00000004737**	***rrbp1a***	**Ribosome binding protein 1 homolog a**	**–**	**–**	*	[95]
**ENSGACG00000005034**	***NAV1 (1 of 2)***		**–**	**–**	*	[95]
**ENSGACG00000006980**	***prkd3***	**Protein kinase D3**	**–**	*	**–**	[95]
**ENSGACG00000009748**	***swap70b***	**SWAP switching B-cell complex subunit 70b**	**–**	*	**–**	[95]
**ENSGACG00000011184**	***tcf7l1b***	**Transcription factor 7-like 1b**	**–**	*	**–**	[95]
**ENSGACG00000011691**			**–**	*	**–**	[95]
**ENSGACG00000012972**	***gorasp2***	**Golgi reassembly stacking protein 2**	**–**	*	*	[95]
**ENSGACG00000013300**	***si:ch211-241e1.3***	**si:ch211-241e1.3**	**–**	**	**–**	[95]
**ENSGACG00000014605**			**–**	*	**–**	[95]
**ENSGACG00000015419**	***cmtm4***	**CKLF-like MARVEL transmembrane domain containing 4**	**–**	**–**	*	[95]
**ENSGACG00000015537**	***cybb***	**Cytochrome b-245, beta polypeptide (chronic granulomatous disease)**	**–**	*	**–**	[95]
**ENSGACG00000015777**	***dmgdh***	**Dimethylglycine dehydrogenase**	**–**	*	*	[95]
**ENSGACG00000017100**	***LRP4***	**Low density lipoprotein receptor-related protein 4**	**–**	*	*	[95]
**ENSGACG00000017390**	***cntln***	**Centlein, centrosomal protein**	**–**	*	*	[95]
**ENSGACG00000019432**			**Yes**	*	*	[95]
**ENSGACG00000019512**	***nxpe3 (7 of 8)***	**Neurexophilin and PC-esterase domain family, member 3**	**–**	**	*	[95]
**ENSGACG00000019730**	***si:dkeyp-34c12.2***	**si:dkeyp-34c12.2**	**–**	*	**–**	[95]
**ENSGACG00000020156**	***si:ch211-55a7.1***	**si:ch211-55a7.1**	**–**	**–**	*	[95]

These are for both Baltic Sea and marine–freshwater pairs of three-spined sticklebacks (regular font), as well as those differentially expressed in salinity and control treatments (bold font).

^a^Gene with SNP loci identified as outliers by BayeScan.

^b^Gene with SNP loci in which allelic distribution is significantly associated with variation in salinity and/or temperature by BAYENV. **: log_10_ (BF) >5; *: log_10_ (BF) > 1.5.

### Local adaptation to the margin: Baltic Sea

The Baltic Sea is a relatively young postglacial ecosystem, formed 6,500 to 9,800 years ago and characterized by steep environmental gradients in salinity, temperature and community composition [69,97]. Earlier reviews have continually drawn attention to the reduced genetic diversity of Baltic Sea organisms compared to populations in the surrounding seas [69,70]. Given that diversity is a prerequisite for adaptation, it may appear that populations in the Baltic Sea may face challenges in adapting to the projected environmental changes, e.g., in salinity and temperature. However, the results of this study suggest the contrary. Earlier evidence for adaptive divergence among Baltic Sea sticklebacks as revealed by a limited number of microsatellite markers was here confirmed to be ubiquitous across the genome. It is likely that such adaptation has arisen from the use of standing genetic variation, since the young age of the Baltic Sea has not allowed much time for new mutations to accumulate. Indeed, the importance of standing genetic variation, as well as the general features of the genomic architecture in ancestral marine sticklebacks, have been demonstrated to play important roles in extensive and parallel genome-wide evolution [59]. However, this has mostly been demonstrated in divergent, isolated population pairs. Our results suggest that the same processes can also occur in the face of gene flow, possibly due to the genomic architecture, which may provide a mechanism for the rapid re-assembly and evolution of multi-locus genotypes in newly colonized freshwater habitats [59,98]. Similar evidence for adaptive divergence is also available from other Baltic Sea species, albeit the scale of sampling and/or marker numbers have often been modest (e.g., [29,31,35,36,38,67,68,70,99]). To this end, the results support the view that in spite of its young age and low species diversity (e.g., [69,97]), the genetic biodiversity stemming from local adaptation to the Baltic Sea seascape may be more widespread than is currently anticipated.

### Methodological considerations

Finally, regarding the methodological considerations, we note that theoretical treatments have shown that sequencing of pooled DNA samples (pool-seq) can be more effective in SNP discovery and can provide more accurate allele frequency estimates than individual sequencing [100]. Nevertheless, pool-seq has its shortcomings: it compromises the ability to conduct certain types of analyses, and certain types of biases and artifacts are possible (e.g., [101-103]). First, information about associations among alleles in different loci is lost, as is the opportunity to estimate linkage disequilibrium. Second, differential amplification of individual samples can create biases in allele frequency estimates [101,103]. Likewise, the assessment of population divergence will be complicated since the sample size (sequencing coverage) available for allele frequency estimation varies among loci. For example, when using PoPoolation tools [104], the accuracy of allele frequency estimation from pooled samples is highly dependent on the sequencing coverage, although the pipeline implements unbiased estimation by considering pool size and sequencing coverage [100]. However, we believe our inference is robust in respect to these potential caveats on the basis of the following. First, the distribution pattern of sequencing coverage for the SNPs we identified was very similar across populations (Additional file 4: Figure S3), suggesting little heterogeneity in sequencing coverage (and by inference, differential amplification of individual samples) across populations. Second, we found that the genome-wide patterns of population differentiation were stable when sequencing coverage varied (Additional file 5: Figure S4). Thus, at least for sequencing coverage, the results and inferences in this paper should be robust. This inference is further supported because the patterns of genomic variability and differentiation observed in this study align well with those seen in other RAD-seq based analyses (e.g., [40,105]), as well as those seen for microsatellite markers in the same set of populations (see above). If large biases in allele frequency estimates were present, such similarities would be unexpected. Comparison of the pool-seq allele frequency estimates with those generated from 30 SNPs genotyped at the individual level verified this conjecture: the correlation between allele frequency estimates across different loci ranged from *r* = 0.75 to *r* = 0.95 depending on the population (Additional file 1: Table S5). Likewise, the correlation between allele frequencies from individual and pooled samples across the 30 loci and all populations was very high (*r* = 0.88, *P* < 2.2 × 10^−16^).

## Conclusions

In summary, we discovered that genome-wide patterns of genetic diversity and differentiation among continuously distributed Baltic Sea three-spined stickleback populations – as assessed from polymorphisms in over 30,000 SNP loci – varied widely across the genome. As such, we identified numerous genomic regions exhibiting signatures of divergent – and to some extent also balancing – selection. We also uncovered strong evidence for substantial genetic differentiation associated with both salinity and temperature gradients, suggesting local adaptation in this high-gene-flow environment. The patterns of genome-wide genetic diversity and differentiation in Baltic Sea three-spined sticklebacks were similar to those observed in earlier studies of marine–freshwater divergence in three-spined sticklebacks, suggesting that the same genetic processes and loci may often underlie adaptation both to freshwater and brackish water environments. Hence, apart from providing strong evidence for genome-wide but heterogeneous genomic divergence driven by local adaptation along an environmental gradient in the post-glacially formed Baltic Sea seascape, our results suggest similarities in genomic signatures of adaptation to freshwater and brackish water environments.

## Methods

The samples used in this study were collected in accordance with the national legislation of the countries concerned. As our samples were derived from wild collected fish, no approval by the Finnish National Animal Experiment Board was required.

### Samples and study sites

Adult three-spined sticklebacks were sampled during the early breeding season of 2009 from ten sites covering most of the Baltic Sea and its opening to the North Sea (Figure 1 and Table 1). The sampling was done with hand seines or minnow traps (mesh size 6 mm). Upon capture, the fish were over-anesthetized with tricaine methanesulfonate (Sigma-Aldrich Co., Saint Louis, United States of America) and stored in 96% ethanol (Altia Oyj, Helsinki, Finland). The study sites and samples are a subset of those used in [47], where more sites were included in analyses with microsatellite markers. The data for average annual salinity and temperature were derived from Table One in [47].

### DNA extraction and restriction site associated DNA sequencing library construction

Whole genomic DNA was extracted from 36 individuals per sampling location, using a NucleoSpin® Tissue kit (Macherey-Nagel, Düren, Germany) following the manufacturer’s protocol. DNA was visualized on a 1% agarose gel to assess quality, and quantified with both a NanoDrop® ND-1000 spectrophotometer and Qubit® fluorometer. Each sample was diluted to 10 ng/μl, re-quantified, and pooled according to sampling location. Each pooled sample was then quantified with both the spectrophotometer and fluorometer and equalized to 10 ng/μl.

RAD library preparation was done by following the protocol detailed by Elshire et al. [106]. Briefly, each of the ten pooled samples was digested with 30 U *Pst*I (New England Biolabs® Inc., Frankfurt, Germany) in 20 μL volumes containing 1× NEBuffer 3 (New England Biolabs® Inc., Frankfurt, Germany) and 1× BSA (New England Biolabs® Inc., Frankfurt, Germany). Reactions were first incubated at 37°C for 2 h, then the temperature was increased to 74°C for 15 min and cooled to 4°C for 10 min. The restriction product was then added to 1× ligation buffer, T4-ligase (New England Biolabs® Inc., Frankfurt, Germany) and an adapter mix containing a common adapter and a barcode adapter unique to each sample. Barcode adapters were selected from the list of 96 sequences provided by Elshire et al. [106]. Then 50 μL ligation reactions were first incubated at 22°C for 1 h, followed by 30 min at 65°C and cooled to 4°C for 10 min. Following purification with a Qiagen Qiaquick kit (QIAGEN, Stockach, Germany), 10 μL of ligation from each population were pooled for library amplification. The library amplification reaction used 10 μL of the pooled ligation product, Phire enzyme, 1× reaction buffer, 1.5 mM MgCl_2_, 10 mM dNTP (New England Biolabs® Inc., Frankfurt, Germany), and 0.5 μM primer mix (see [106] for primer sequences) in 50 μL volumes. PCR was initiated at 72°C for 1 min, then raised to 95°C for an additional 30 s, followed by 18 cycles of 95°C for 30 s, 65°C for 30 s and 72°C for 20 s. A final extension at 72°C for 5 min concluded the reaction. Products were visualized on 2.5% MetaPhor low-melt agarose gel, and fragments of 250 to 350 bp were excised after running for 2 h at 80 V and cleaned with QIAquick® Gel Extraction Kit (QIAGEN, Stockach, Germany) according to the manufacturer’s protocol.

### Sequencing, data processing, and alignment

Barcoded RAD samples were sequenced on one lane of the Illumina HiSeq2000 platform with a 100-bp single-end strategy by BGI Hongkong Co, Limited. Using the FastX toolkit [107], all raw reads were end-trimmed to a length of 90 bp, and reads containing one or more bases with a Phred quality score below 10 or more than 5% of the positions below 20 were discarded.

Quality filtered reads from each sample were aligned to the three-spined stickleback genome (release-73, Ensembl) separately using BWA 0.7.5a [108]. The maximum edit distance was two and the maximum number of alignments for each read was one. The mapping results in SAM format were converted into BAM format using SAMtools 0.1.18 [109] and filtered for a minimum mapping quality of 20. BAM files were then converted into mpileup format using SAMtools 0.1.18 with a maximum of 1,000 reads at a given position per BAM file.

### Estimation of genome-wide genetic variation and differentiation

To characterize genome-wide patterns of genetic variation and population differentiation, nucleotide diversity (Tajima’s *π*), population mutation rate (Watterson’s theta, *θ*_*W*_) and Tajima’s *D* were estimated using PoPoolation 1.2.2 [104]. In addition, the fixation index (*F*_ST_) values for each pairwise comparison were estimated using PoPoolation2 [110], by implementing a number of stringent criteria to define genomic sites for analysis across the entire genome. Since the accuracy of allele frequency estimation in the sequencing of pooled individuals is highly dependent on sequence coverage, we used high sequence coverage and large sliding windows (see below), as they are expected to increase the accuracy of the above-mentioned population genetic parameters by decreasing stochastic error [104]. To estimate *π* and *θ*_*W*_, all genomic sites subjected to analysis were required to have a minimum minor allele count of 2 and coverage between 10 and 500 for each population, as well as a minimum minor allele count of 4 and coverage between 20 and 1,000 across all the 10 populations. Since Tajima’s *D* is sensitive to variation in coverage [104], it was only calculated for genomic sites with a coverage of 36 for each population and for alleles with a coverage of 72 across all the 10 populations. *F*_ST_ values for each pairwise comparison were estimated for genomic sites with a minimum minor allele count of 4 across all the 10 populations and coverage between 10 and 500 within each population. To make this study comparable to other population genomic studies of marine and/or freshwater three-spined sticklebacks [57,58,60,65], a non-overlapping 100-kb sliding window was used for estimating the above-mentioned population genetic parameters across the entire genome with a minimum base Phred quality of 20 for the analyzed genomic sites. Patterns of genomic variation as reflected in *F*_ST_, Tajima’s *π*, *θ*_*W*_ and Tajima’s *D* were visualized using Circos [111].

### Detection of selection footprints

To identify genes likely to be differentiated as a result of selection, a subset of SNPs were identified using PoPoolation2 with stringent criteria: a minimum minor allele count of 6, and coverage between 36 and 500 across all the 10 populations. Two independent methods were employed to identify selection. First, pairwise *F*_ST_ values for each of the subsets of SNPs were calculated between populations using PoPoolation2. SNPs falling into the upper 0.5% tails of at least 5 of the 45 pairwise *F*_ST_ comparisons were identified as potentially differentiated loci, following an empirical outlier detection approach [58,112]. Second, to verify whether this empirical approach is reliable, a simulated multi-locus dataset of the subset of SNPs was exported from PoPoolation2, and BayeScan 2.1 [113] was used for estimating the posterior probability that a given locus is affected by selection. Briefly, prior odds of 100 were used for identifying the top candidates of the selected loci and a total of 55,000 reversible-jump Markov chain Monte Carlo chains were run with a thinning interval of 10, following 20 pilot runs of 5,000 iterations each, and a burn-in length of 50,000. Loci were considered under selection with a FDR of 0.05. Only SNPs that were identified as outliers by both of the two above-mentioned approaches were considered as truly differentiated loci.

### Detection of genetic differentiation associated with environmental parameters

To test for association between genetic differentiation and environmental parameters, a Bayesian approach as implemented in BAYENV [73] was applied to the subset of SNPs identified by PoPoolation2. The Bayesian approach takes into account the effect of population structure, using a covariance matrix based on neutral markers to control for demographic effects when testing for correlations between environmental and genetic differentiation [73]. To do so, a neutral covariance matrix based on the neutral SNPs (as revealed by outlier tests; see below) was first estimated, and then two environmental parameters (viz. average annual salinity and average annual temperature; Table 1) were tested for association with genetic variation. Each environmental parameter was standardized by subtracting the mean and dividing by the standard deviation of the parameter across all sites. To verify that the results were not sensitive to stochastic errors, three independent runs with different random seeds were run.

### SNP annotation and gene ontology analysis

The three-spined stickleback genome annotations were downloaded from Ensembl (release-73). BEDTools 2.17.0 [114] was used for annotation of the subset of SNPs identified by PoPoolation2 to characterize whether the SNPs were located within a gene. GO terms for the three-spined stickleback genes were retrieved with BioMart [115] from Ensembl. A GO enrichment analysis was conducted to test if certain gene classes were over- or underrepresented among genes harboring outlier loci compared to the genes harboring the remaining neutral SNPs, using GOSSIP [116].

### Characterization of population structure

The population structure was characterized on the basis of the pairwise *F*_ST_ matrices among populations estimated using the subset of SNPs identified by PoPoolation2. To visualize the multi-locus patterns of population differentiation, a PCO plot was generated using the R package labdsv [117] based on average *F*_ST_ values. To examine further the patterns of population differentiation, a neighbor-joining tree based on *F*_ST_ values [118] was constructed using the simulated multi-locus dataset of the subset of SNPs identified by PoPoolation2 with 1,000 bootstrap replicates in Populations 1.2.32 [119] software.

To compare the patterns of genetic variability and differentiation in SNP markers with those in microsatellite markers, we retrieved data from 40 microsatellite loci genotyped for these same populations [47]. A simple correlation analysis was used to compare genetic variability (average heterozygosity) across populations, whereas the patterns of population differentiation (as reflected in pairwise *F*_ST_ estimates) were compared with a Mantel’s test. Tests for isolation by distance were conducted with a Mantel’s test using linearized *F*_ST_ values [*F*_ST_ / (1 – *F*_ST_)] and log-transformed geographic distances separating sampling locations.

### Allele frequency validation

To validate estimates of allelic frequency from the pool-seq data, we genotyped a subset of 30 SNPs from each of the individual fish used for the pooled DNA analyses using the iPlex Gold® assay on the MassARRAY® platform (Sequenom) system. This genotyping was performed by the Technology Centre of the Institute for Molecular Medicine Finland at the University of Helsinki. Allele frequencies from this data were estimated with a custom Perl script and compared to estimates from pooled data as obtained using the procedures above.

### Data accessibility statement

Sequences underlying this study have been deposited in NCBI’s Sequence Read Archive and accession numbers are SRR1596320, SRR1596321, SRR1596322, SRR1596323, SRR1596324, SRR1596325, SRR1596326, SRR1596327, SRR1596328, and SRR1596329.

## Additional files

Additional file 1: Table S1. Chromosome length (bp), number of *Pst*I restriction sites and RAD-seq read mapped on each chromosome in each population. **Table S2.** Number of SNPs identified on each chromosome in each population with PoPoolation. **Table S3.** Nucleotide diversity (*π* / *θ*_*W*_) of each chromosome in each population based on polymorphic loci shown in Table S2. **Table S4.** Candidate genes associated with adaptation. Outlier: Gene included outlier loci. Salinity: Gene included SNPs for which allele frequency was associated with salinity variation of sampling site. Temperature: Gene included SNPs for which allele frequency was associated with annual temperature variation of sampling site. *: Gene included SNPs had log_10_ (Bayes factor) > 5. **Table S5.** Comparison of allele frequencies between individual pool-seq samples in ten different populations at 30 SNP loci. *r* is the correlation in allele frequencies across the loci in a given population. Additional file 2: Figure S1. Correlations between number of *Pst*I restriction sites **(a)**, mapped reads **(b)**, and number of SNPs on each chromosome **(c)** against chromosome length in the population COP. Mbp, megabase pair. Additional file 3: Figure S2. Genome-wide distribution of genetic variation in each of the ten study populations. Chromosomes are labeled in black Roman numerals and represented as grey and black blocks. Additional file 4: Figure S3. Sequencing coverage for SNPs identified in each of the study populations. Additional file 5: Figure S4. Genome-wide distribution of genetic differentiation across all of the 10 three-spined populations in the Baltic Sea with variable sequencing coverage. Red line: sequencing coverage between 10 and 500; green line: sequencing coverage between 10 and 360; blue line: sequencing coverage between 36 and 360.

## Abbreviations

BF: Bayes factor
bp: base pair
BSA: bovine serum albumin
FDR: false discovery rate
GO: gene ontology
kb: kilobase
PCoA: principal coordinates analysis
PCR: polymerase chain reaction
RAD-seq: restriction site associated DNA sequencing
SNP: single nucleotide polymorphism
SE: standard error
