# Supplementary figures and images for: Population genomic evidence for adaptive differentiation in Baltic Sea three-spined sticklebacks
Source: BMC Biol. 2015 Mar 24;13:19. doi: 10.1186/s12915-015-0130-8 (PMC4410466; doi:10.1186/s12915-015-0130-8)

**(a)**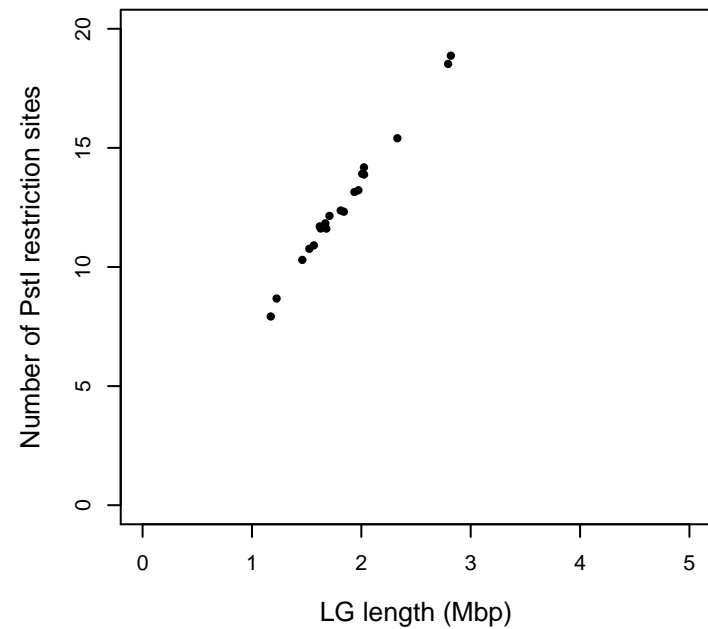**(b)**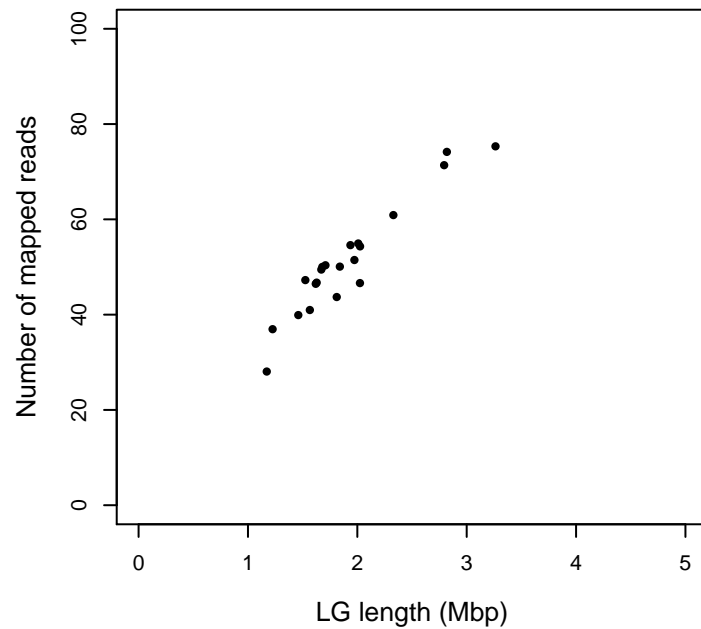**(c)**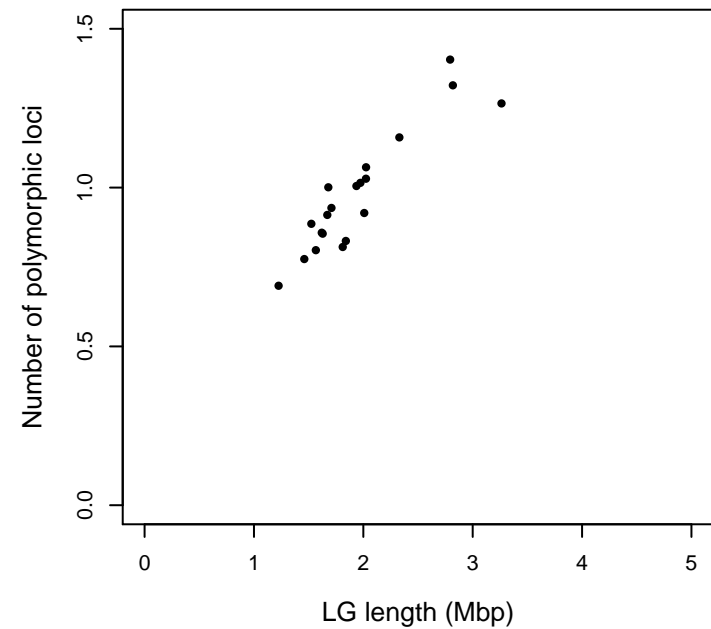

Supplement: Additional file 2: Figure S1. — Correlations between number of PstI restriction sites (a), mapped reads (b), and number of SNPs on each chromosome (c) against chromosome length in the population COP. Mbp, megabase pair. [file 12915_2015_130_MOESM2_ESM.pdf]

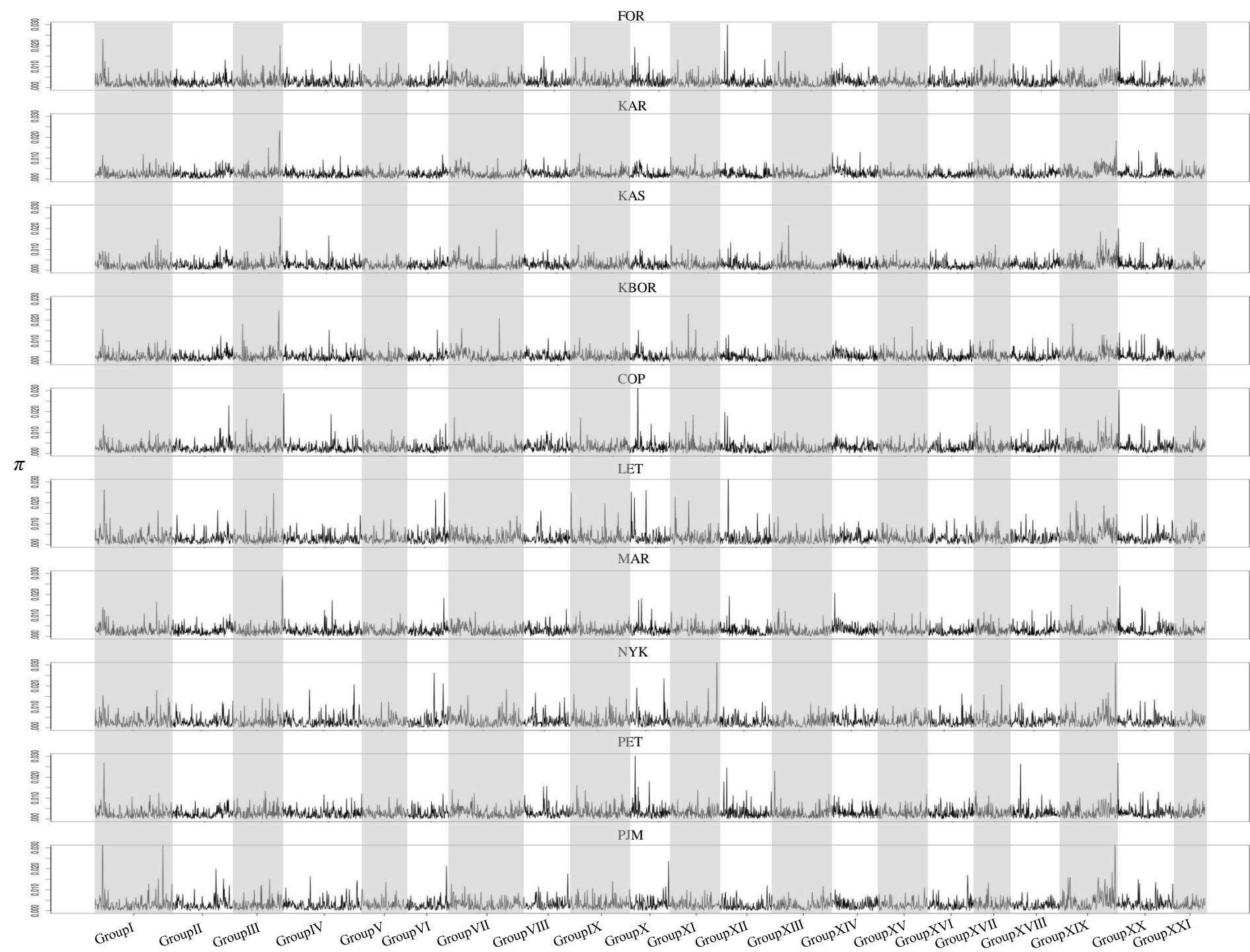

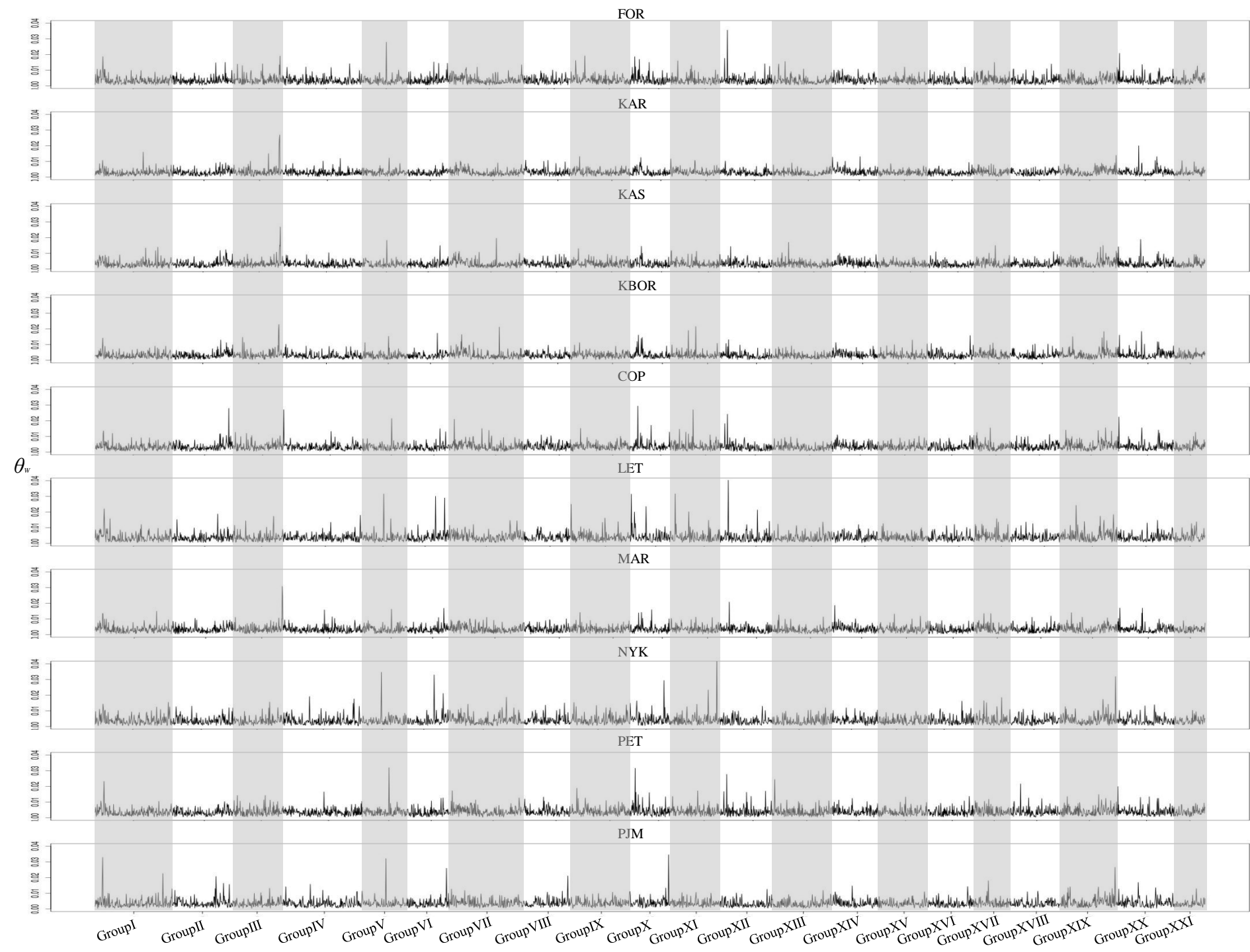

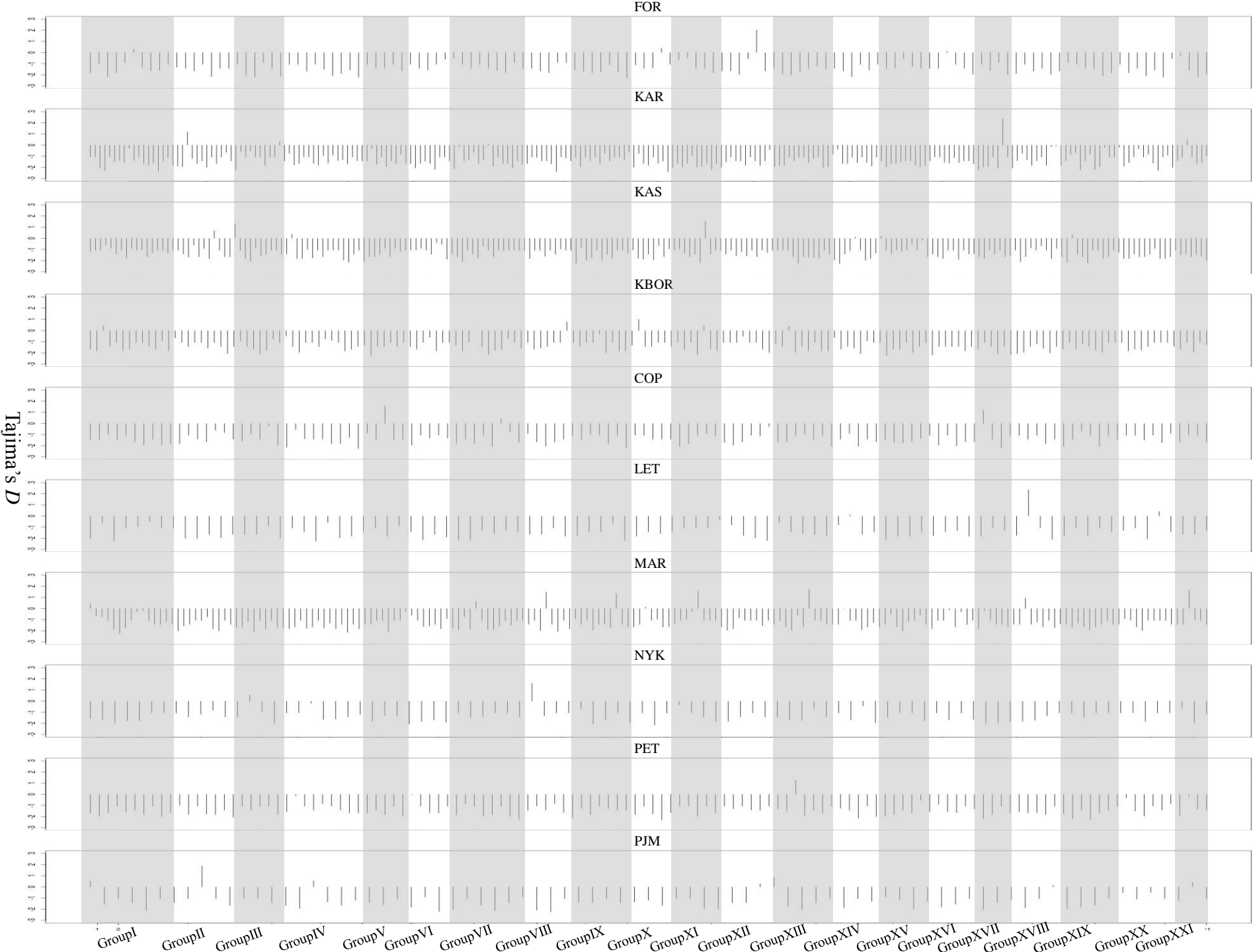

Supplement: Additional file 3: Figure S2. — Genome-wide distribution of genetic variation in each of the ten study populations. Chromosomes are labeled in black Roman numerals and represented as grey and black blocks. [file 12915_2015_130_MOESM3_ESM.pdf]

**COP**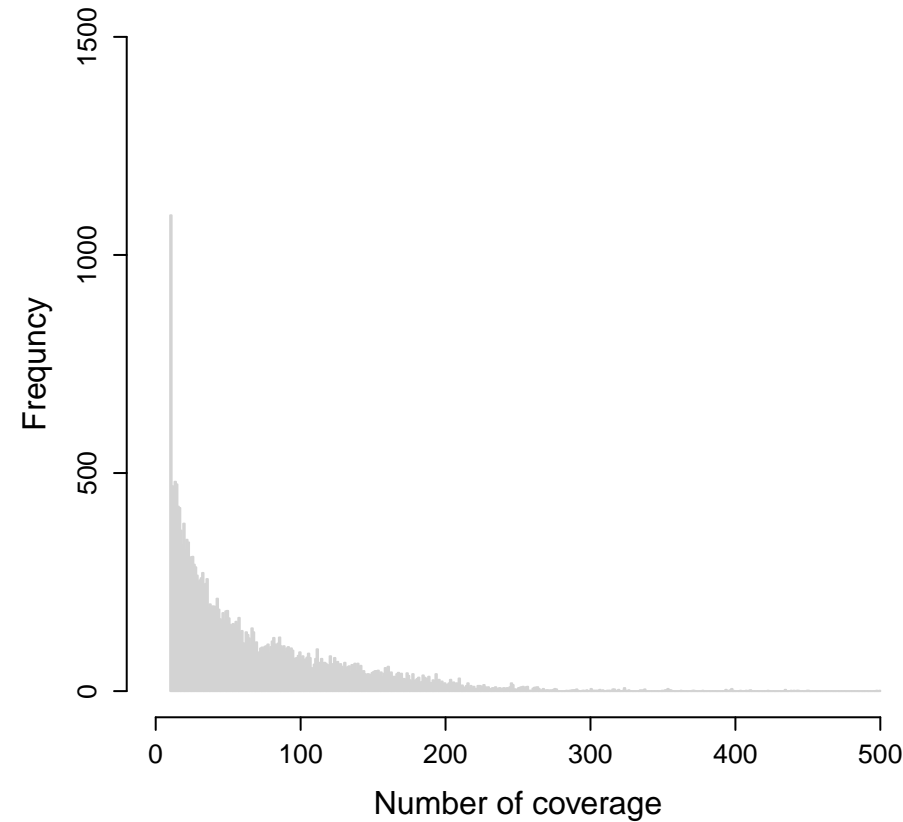**FOR**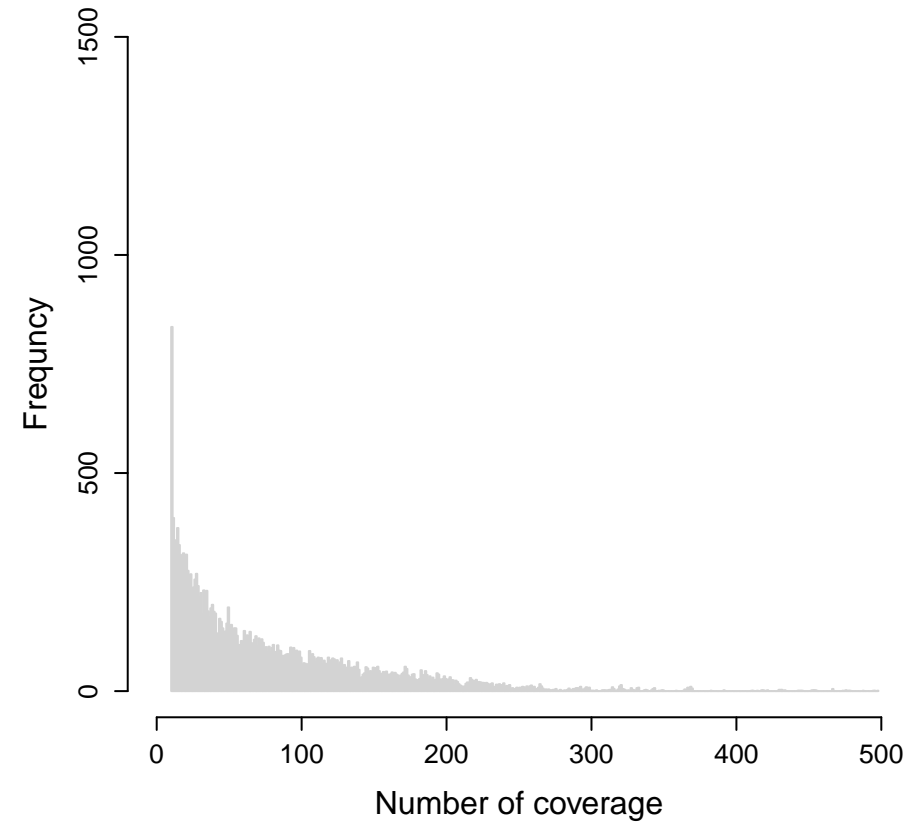**KAR**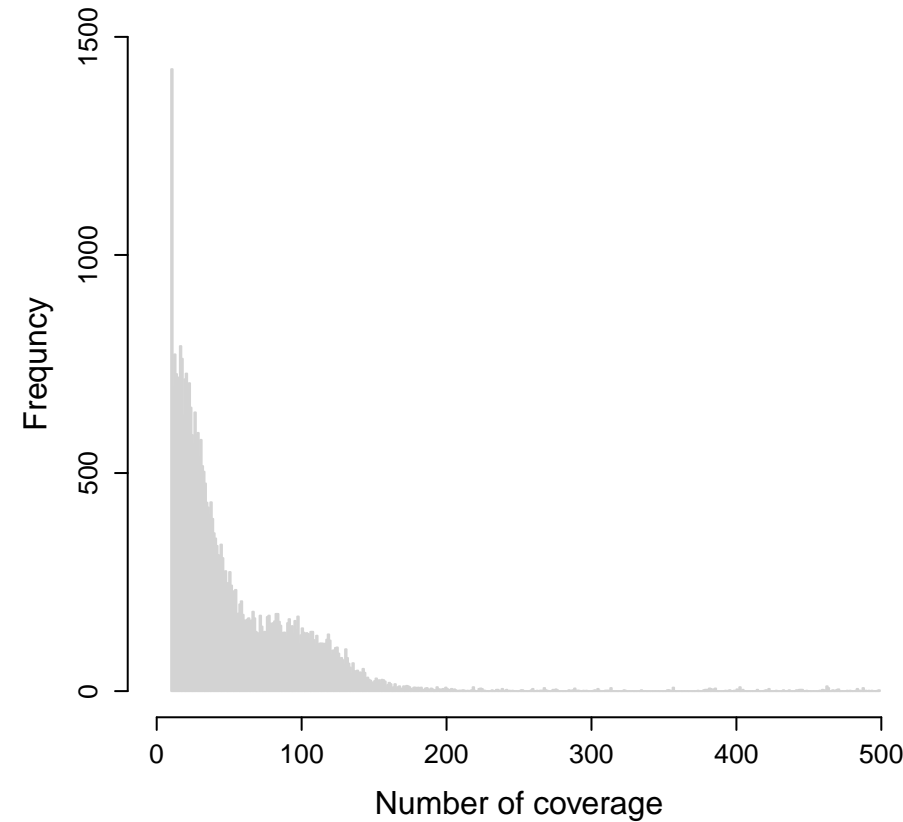**KAS**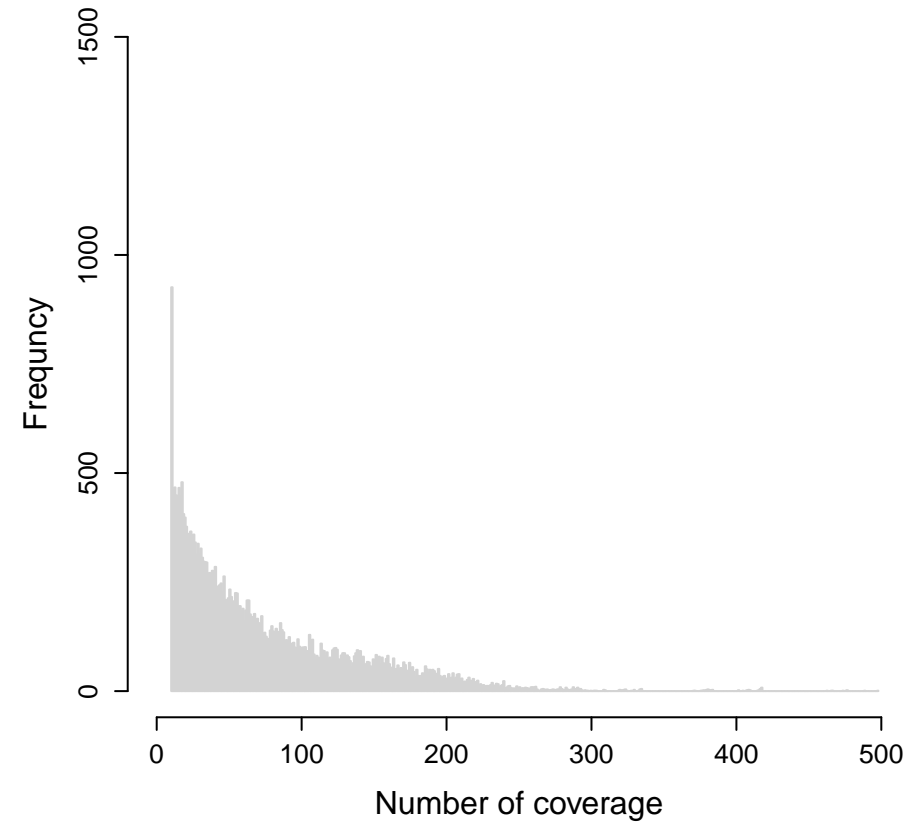**KBOR**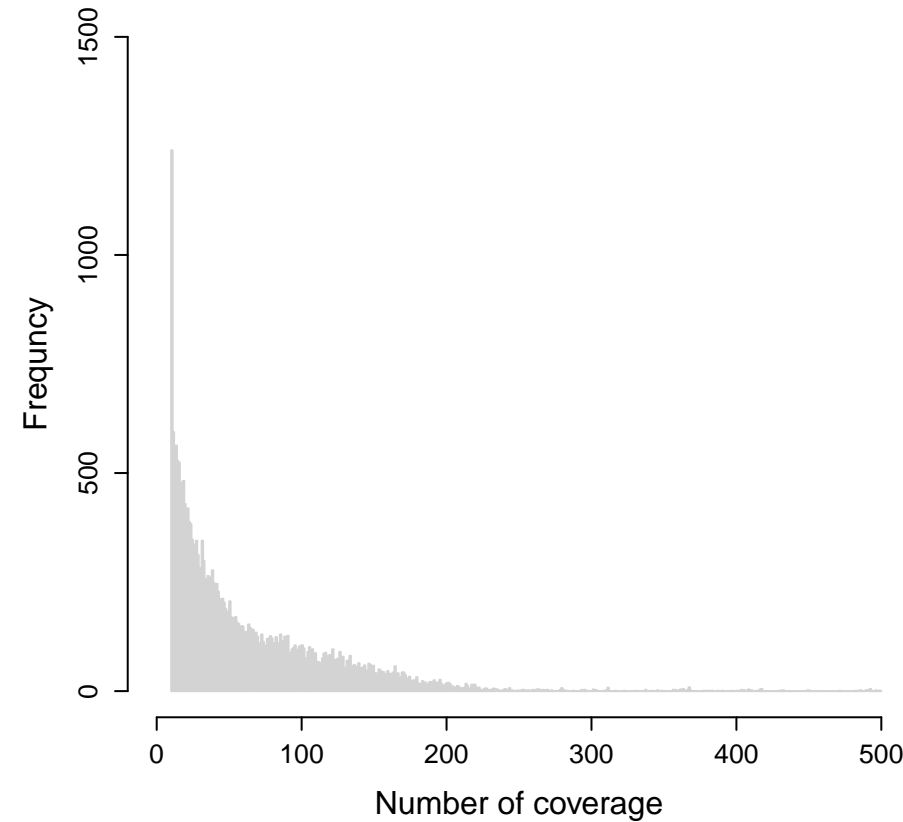**LET**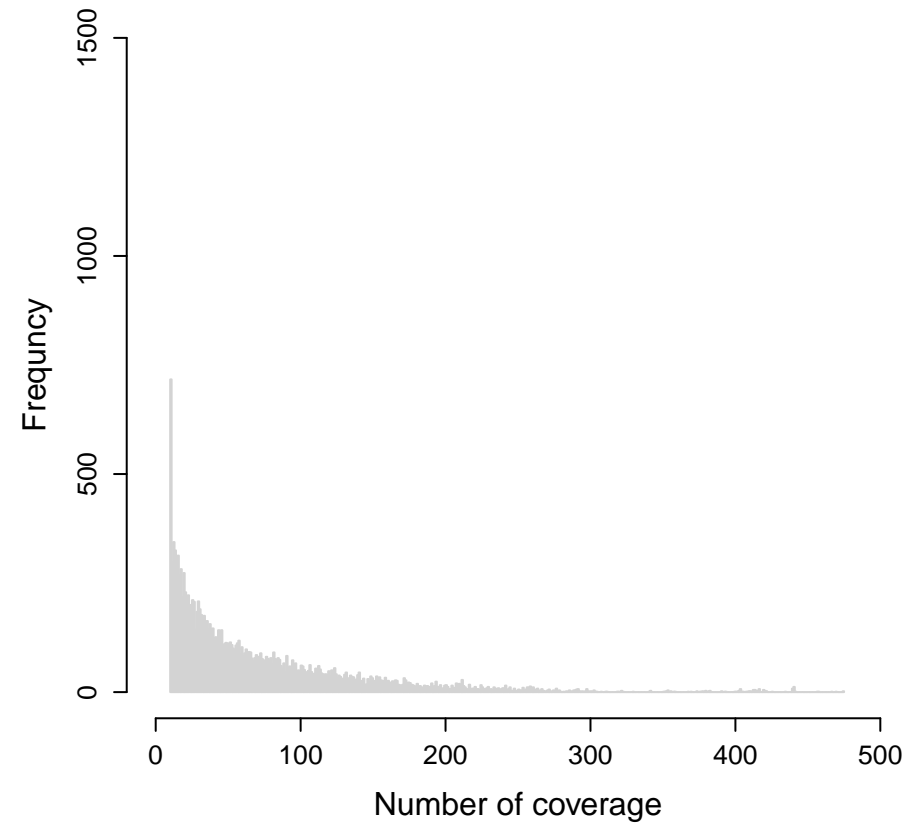**MAR**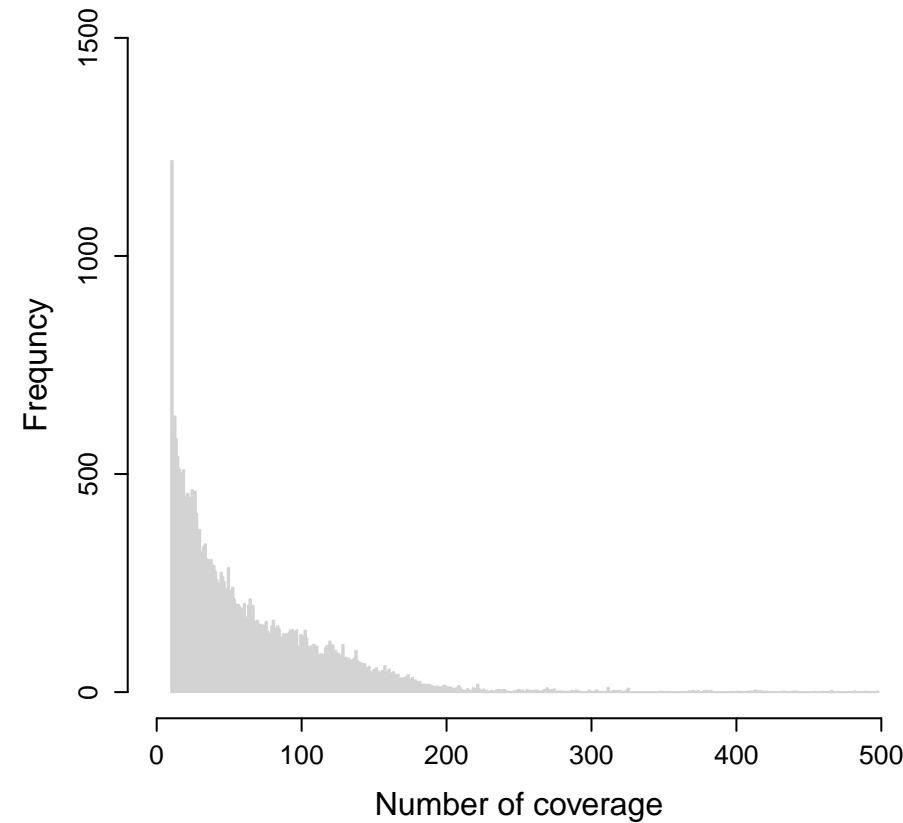**NYK**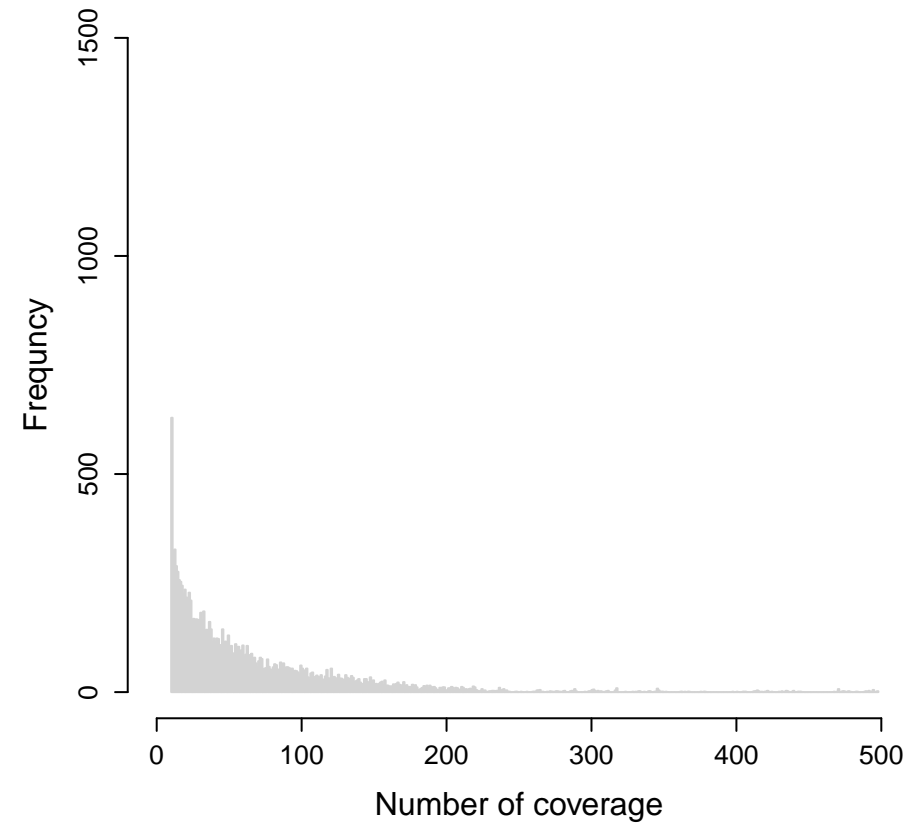**PET**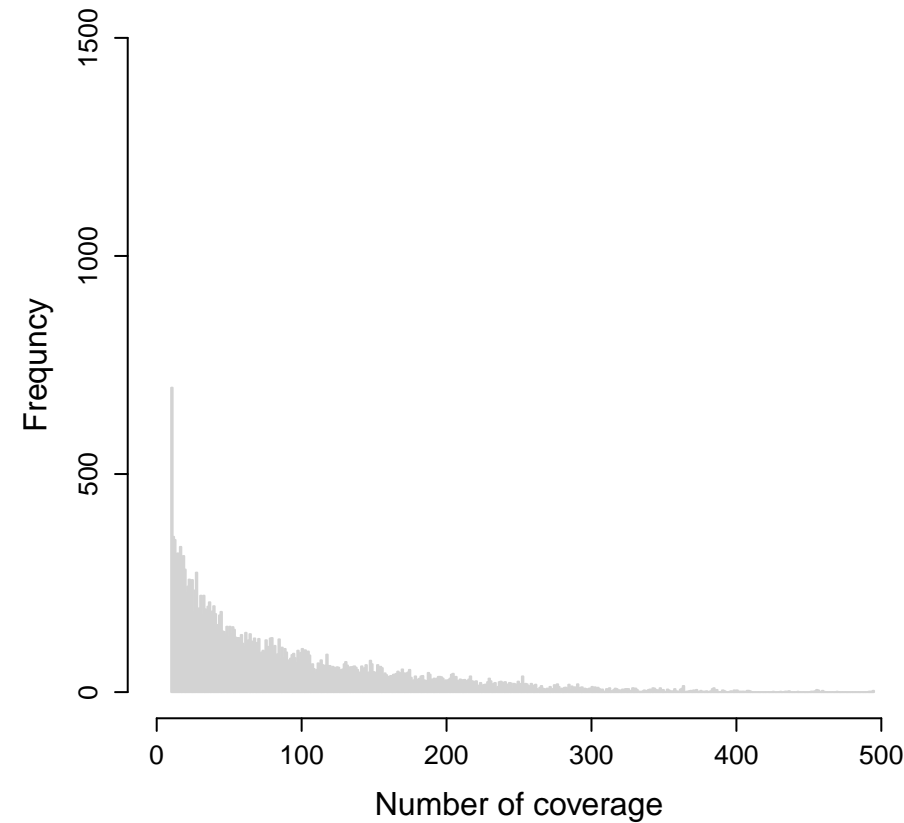**PJM**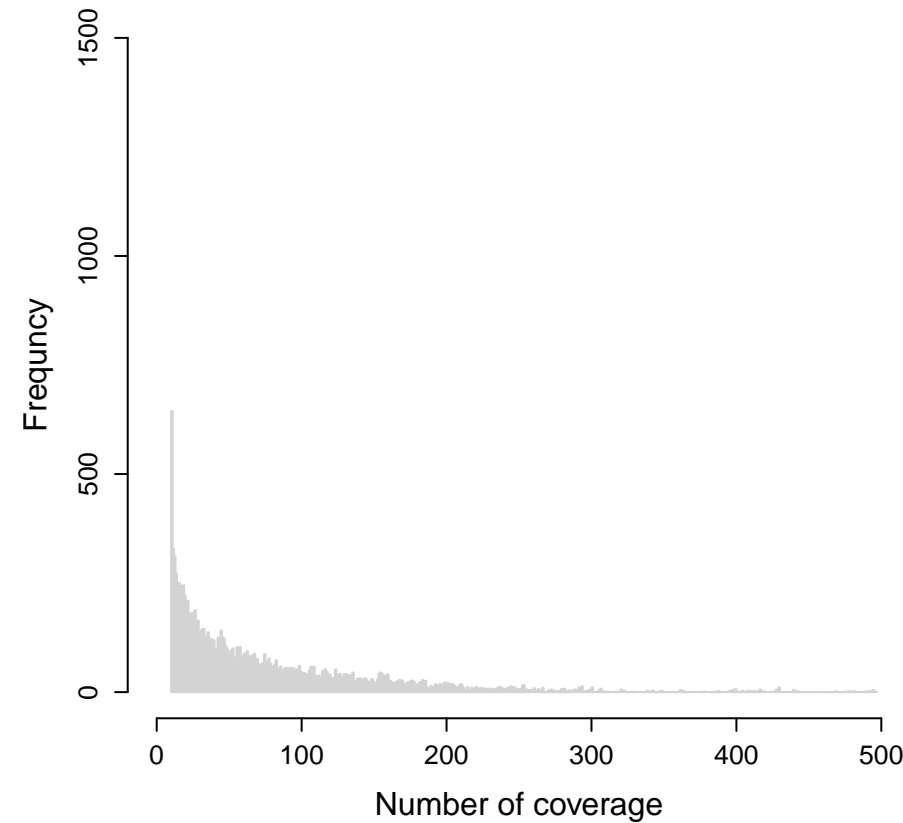

Supplement: Additional file 4: Figure S3. — Sequencing coverage for SNPs identified in each of the study populations. [file 12915_2015_130_MOESM4_ESM.pdf]

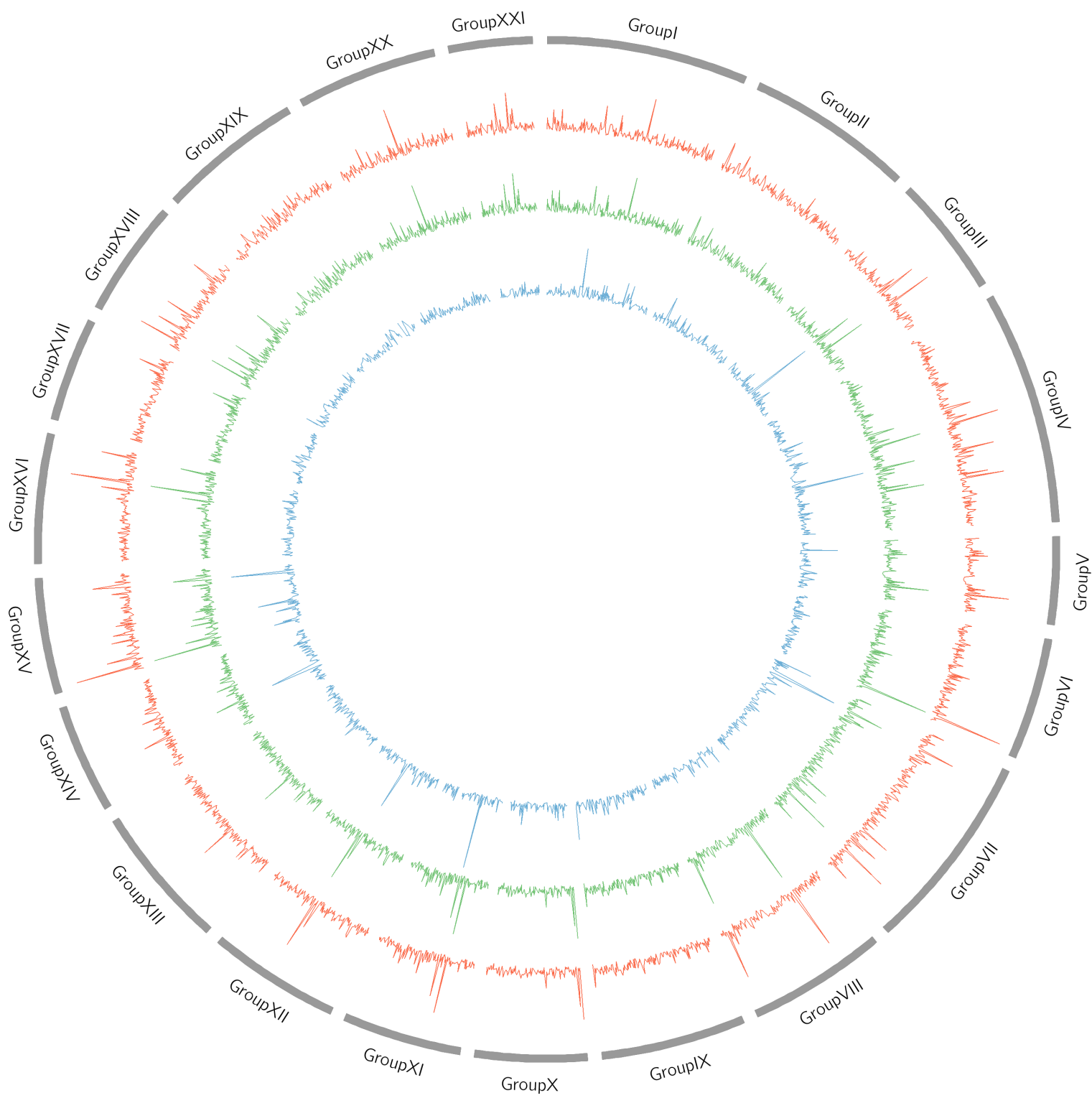

Supplement: Additional file 5: Figure S4. — Genome-wide distribution of genetic differentiation across all of the 10 three-spined populations in the Baltic Sea with variable sequencing coverage. Red line: sequencing coverage between 10 and 500; green line: sequencing coverage between 10 and 360; blue line: sequencing coverage between 36 and 360. [file 12915_2015_130_MOESM5_ESM.pdf]
